# Synthesis of Cystine-Stabilised Dicarba Conotoxin EpI: Ring-Closing Metathesis of Sidechain Deprotected, Sulfide-Rich Sequences

**DOI:** 10.3390/md21070390

**Published:** 2023-06-29

**Authors:** Amy L. Thomson, Andrea J. Robinson, Alessia Belgi

**Affiliations:** School of Chemistry, Monash University, Clayton, VIC 3800, Australia; amy.thomson1@monash.edu (A.L.T.); andrea.robinson@monash.edu (A.J.R.)

**Keywords:** olefin metathesis, cysteine, methionine, disulfide replacement, unprotected peptides, dicarba peptides

## Abstract

Recombinant peptide synthesis allows for large-scale production of peptides with therapeutic potential. However, access to dicarba peptidomimetics via sidechain-deprotected sequences becomes challenging with exposed Lewis basicity presented by amine and sulfur-containing residues. Presented here is a combination of strategies which can be used to deactivate coordinative residues and achieve high-yielding Ru-catalyzed ring-closing metathesis. The chemistry is exemplified using α-conotoxin EpI, a native bicyclic disulfide-containing sequence isolated from the marine conesnail *Conus episcopatus*. Replacement of the loop I disulfide with *E/Z*–dicarba bridges was achieved with high conversion via solution-phase ring-closing metathesis of the unprotected linear peptide after simple chemoselective oxidation and ion-exchange masking of problematic functionality. Metathesis was also attempted in green solvent choices to further improve the sustainability of dicarba peptide synthesis.

## 1. Introduction

The annual cost of pain in Australia, including healthcare provision, productivity costs, and loss of income, is 139 billion AUD and is estimated to rise to 215 billion AUD by 2050 [1]. Meanwhile, pain management remains reliant on morphine-based drugs despite problems of tolerance and addiction, as well as associated side effects, including constipation, respiratory suppression, and fatigue [2,3]. There is, therefore, high demand for new lead compounds based on alternative therapeutic targets.

Ion channels play a pivotal role in the transmission of pain signals. Peptides capable of modulating the activity of pain-inducing ion channels have been identified in the venom of predatory marine cone snails [4]. Conotoxins are predominantly small disulfide-rich peptides comprised of 10 to 40 residues and 1 to 4 disulfide bridges.

Analgesic conotoxins have advantages over morphine-based drugs as they do not have a profile associated with tolerance and abuse, and they have emerged as useful leads for the development of novel analgesics for the amelioration of chronic pain. Towards this end, MVIIA, an α-conotoxin isolated from the venom of the cone snail *Conus magus*, is able to block N-type calcium channels (Ca_v_2.2) [5,6] and was approved and released into the market for the management of chronic pain (Ziconotide, Prialt^®^) [7]. However, as it is unable to cross the blood–brain barrier, its administration is limited to intrathecal infusion, making Prialt^®^ the treatment of choice only when all other therapeutic options are exhausted [8,9]. Another conotoxin, Vc1.1, from the species *Conus victoriae* [10], was evaluated in neuropathic pain trials by Metabolic Pharmaceuticals Pty Ltd., currently a subsidiary of PolyNovo, Melbourne, Australia. After completion of Phase 2A, however, the clinical trial was discontinued due to poor specificity and lack of efficacy at the then postulated molecular target, α9α10 nicotinic acetylcholine receptors (nAChRs) [11]. Importantly, no significant side effects were recorded from the trials. Recently other molecular targets, with a particular focus on the GABA_B_ receptor, have been identified for Vc1.1 and other structurally related α-conotoxins [12,13,14,15,16]. In light of this new evidence, there has been a developing interest in conotoxins, and particularly α-conotoxins, to understand their mode of action and to overcome some of their shortfalls. α-Conotoxins usually contain 12 to 20 residues and four cysteines with oxidised connectivity Cys1-Cys3/Cys2-Cys4 to obtain a globular fold [17]. Their small size, in particular, makes them perfect candidates in clinical studies as synthetic analogues are easily synthesised using automated solid phase peptide synthesis (SPPS).

While the native peptides show promise as rapid-acting and potent receptor signalling modulators, some of their accompanying properties, such as limited bioavailability, disulfide scrambling [18,19,20] and susceptibility to reduction [21], can limit their clinical use. In fact, the bioactive conformations of conotoxins are preserved by their disulfide frameworks, and the in vivo reduction of the disulfide bridges can lead to inactive linear sequences and incorrectly recycled inactive topoisomers.

Considerable research effort has been dedicated to the synthesis of α-conotoxin analogues to achieve improved in vivo stability, enhanced potency and improved selectivity at the cognate receptors. Dicarba replacement of the metabolically unstable disulfide bridges provides protection against in vivo reduction and disulfide scrambling and also preserves the ring size formed by the native disulfide bridge in the bioactive conformation. In terms of receptor selectivity, Robinson et al. replaced each of the disulfide bridges in α-conotoxins Vc1.1 and RgIA with unsaturated dicarba bridges using olefin metathesis [22]. The resultant dicarba Vc1.1 and dicarba RgIA regioisomers possessed receptor selectivity for either the nAChR or GABA_B_ receptor: When Cys1-Cys3 was replaced with a dicarba bridge, the *cis*- and *trans*-dicarba analogues were selectively active at the GABA_B_ receptor; conversely, when the Cys2-Cys4 was replaced with a dicarba bridge, the analogues were only active at selected nAChRs [22,23].

In addition to improving the pharmacological profile of α-conotoxins, the dicarba geometric isomers provide additional information about the bioactive conformation of the disulfide bridge and provide insight into the native peptide’s mechanism of action. This was previously shown in a study investigating dicarba analogues of human insulin [24].

Synthesis of dicarba analogues has been achieved in many peptide sequences with the aid of solid support. Microwave irradiation and the insertion of turn-inducing pseudoproline residues between the olefinic residues overcome issues of peptide aggregation, contributing to an improved or even quantitative yield of the ring-closing metathesis (RCM) product [25,26,27]. However, the need for multiple orthogonal protecting groups, high excess of reagents, and unsafe organic solvents, such as DMF and DCM, limit the scalability and sustainability of this approach.

More recently, steps have been taken towards achieving a greener synthesis of dicarba peptides. The recombinant synthesis of linear peptide sequences has been modified to allow for the inclusion of olefinic residues required for ring-closing metathesis [28,29]. However, the translation of metathesis chemistry to these substrates requires the mitigation of catalyst-coordinating functional groups within sidechain deprotected peptide sequences.

Masking of Brønsted bases, such as the peptide *N*-terminus and basic amino acids (e.g., lysine and arginine), as ammonium salts [30] protects against Ru-alkylidene catalyst poisoning and has allowed the cyclisation of a wide range of unprotected peptides in solution [31,32,33]. Additionally, the negation of the deleterious catalyst-coordinating effects of sulfur-containing residues, cysteine and methionine, was also demonstrated in unprotected peptides in solution [33]. Cysteine residues were masked through oxidation to the disulfide bridge, and similarly, the methionine thioether functionality was masked through oxidation to the sulfoxide functionality. Importantly, both of these masking oxidation reactions are reversible and biocompatible. The thiol masking chemistry was used on the naturally occurring peptide Vc1.1 to generate [Cys1-Cys3]-dicarba Vc1.1, whereas the thioether masking chemistry was developed on a fragment of the A-chain of human insulin engineered to bear a non-native methionine residue. In the present work, the chemistries developed for the RCM on unprotected peptides were combined to generate dicarba analogues of the α-conotoxin EpI, a naturally occurring peptide bearing two interlocked disulfide bridges, a basic arginine residue, and a sulfurous methionine residue.

α-Conotoxin EpI is one of many peptides isolated from the venom of *Conus episcopatus*, a worm-hunting marine conesnail found in the tropical Indo-West Pacific Ocean. Its interlocked cystine bridged sequence, bearing loop sizes of four and seven, basic residues at the *N*-terminus and Arg7, and the coordinative potential of the thioether residing within Met10, provide a challenging substrate for protecting group-free RCM. The loop I sequence of EpI, GCCSDPRC, is consistent with other α-conotoxins sequences known to provide potent in vivo analgesia (e.g., Rg1A, Vc1.1) [34]. This disulfide bridge formed from Cys residues at positions two and eight can be replaced with dicarba mimetics to provide potent analgesic analogues possessing increased metabolic stability and receptor target selectivity [22,23,35]. In this respect, the EpI sequence has every residue known to compromise Ru-alkylidene catalysed metathesis and therefore provides an unprecedented test for our devised methodology. The generation of novel dicarba analogues of EpI may also shed light on the elusive mechanism of action of several analgesic α-conotoxins.

## 2. Results and Discussion 

EpI, a 16-residue native peptide bearing post-translational sulfation on Tyr15 as well as *C*-terminal amidation, is reported to target neuronal nAChR α3β2 and α3β4 subtypes [36,37]; its activity at the GABA_B_ receptor is unknown. Modest differences in activity have been reported between sulfotyrosine and tyrosine variants [38] and because installation of a dicarba bridge at loop I of structurally related α-conotoxin peptides, such as Vc1.1 and RgIA, is known to abolish nAChR activity [22,23], nor-sulfotyrosine analogues were employed in this study.

**Scheme 1:**
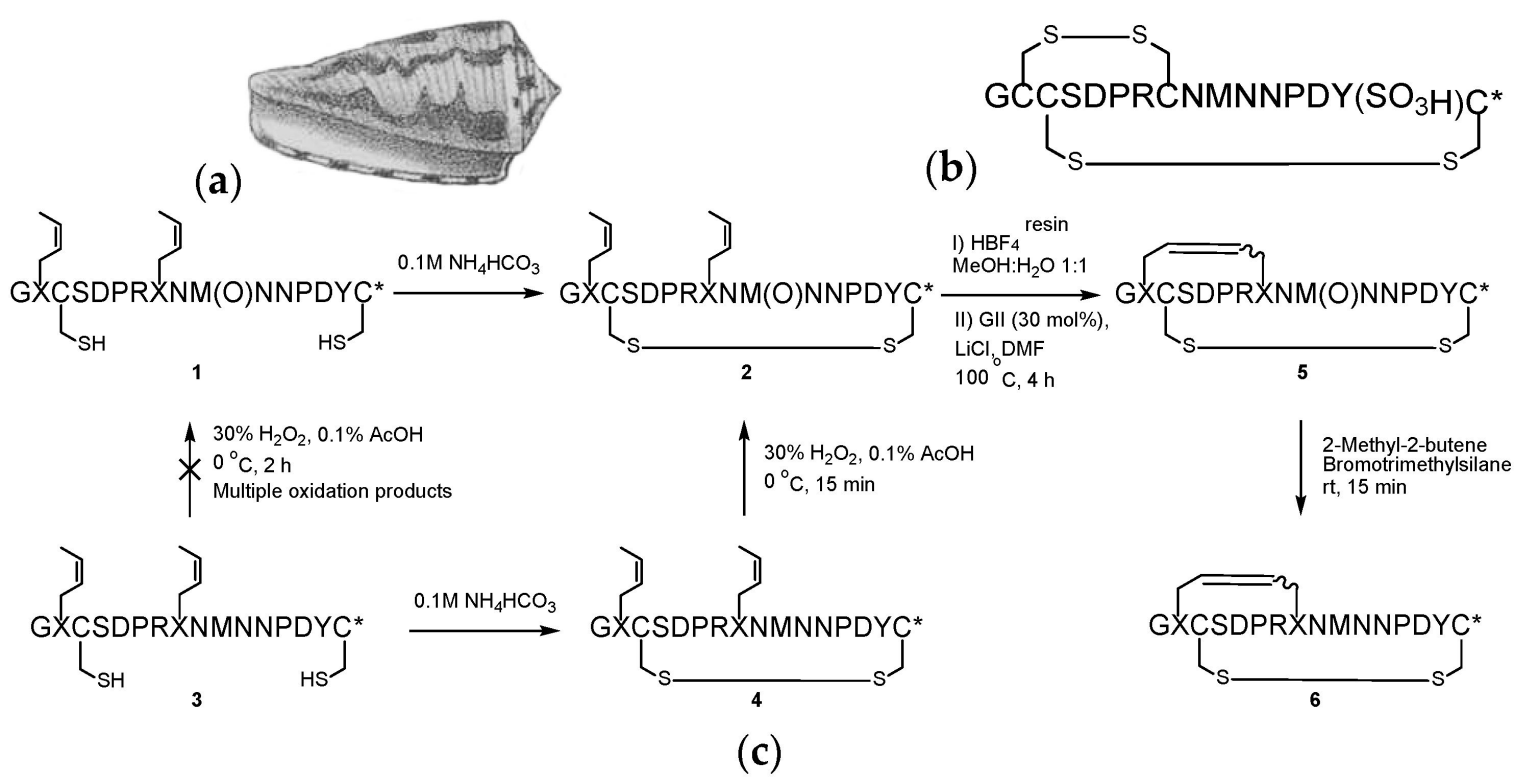
(**a**) *Conus magus*; (**b**) conotoxin EpI; and (**c**) protecting group-free synthesis of *c*[2,8]-dicarba-α-conotoxin EpI **6** (* signifies *C*-terminal amide).

For the loop I disulfide to be replaced with a dicarba bridge, a pair of metathesis-active residues are required in the linear sequence in positions two and eight. Non-terminal *Z*-crotyl glycine was selected to minimise Ru-alkylidene catalyst decomposition and avoid catalytic cycling via the Ru-methylidene species [39]. Linear sequence [Met(O)10, Tyr15]EpI (**1**) was synthesised using standard solid-phase peptide synthesis with Fmoc-protected amino acids (Scheme 1). Inclusion of commercially available Fmoc-protected L-methionine-DL-sulfoxide provides protection of the native position 10 methionine as the sulfoxide, Met(O). The linear sequence was constructed on Rink amide AM resin on a 0.1 mmol scale, yielding peptide **1** as the major product after TFA-mediated cleavage and analysis by RP-HPLC and MS.

Peptide **1** was then subject to buffered aerial oxidation to form the monocyclic disulfide peptide **2** to reduce the Lewis basicity of the two cysteine residues in preparation for the catalysis step (Scheme 1). Oxidation achieves full conversion to peptide **2**, which is subsequently purified by preparative RP-HPLC.

In the future, large-scale synthesis of therapeutic dicarba peptides may be best achieved through solution-phase RCM of recombinantly derived linear sequences. Despite advances in expanding the genetic code to accommodate for the inclusion of non-standard amino acids [40,41,42], recombinant synthesis of peptides containing Met(O) would require complicated biochemical engineering of tRNA constructs and would likely be hampered by post-translational modifications that could reduce it back to methionine. Thus, in a convergent synthesis towards metathesis substrate **2**, recombinantly accessible precursor [Met10, Tyr15]EpI (**3**) (Scheme 1) was synthesised via SPPS on Rink amide AM resin on a 0.1 mmol scale, yielding the target peptide as the major species after TFA-mediated cleavage and analysis by RP-HPLC and MS.

In order to achieve chemoselective oxidation of Met to Met(O), the Cys residues were oxidised to pre-install the required loop II disulfide bond prior to sulfoxide production. Buffered aerial oxidation of **3** provides clean conversion to monocyclic **4** (Scheme 1). Following a procedure adapted from Petitdemange et al. [43], the Met to Met(O) conversion of peptide **4** chemoselectively proceeds to the desired product **2** with full conversion within 15 min of exposure to H_2_O_2_ (Scheme 1). Thus, a convergent synthesis method to peptide **2** is achieved that is capable of proceeding via a greener route from the recombinantly accessible starting material.

Alternatively, attempts at the oxidation of Met to Met(O) in the presence of free Cys residues were unsuccessful. Exposure of peptide **3** to H_2_O_2_ achieves desired product **1** alongside additional species originating from oxidation sidereactions of the free cysteine residues: the disulfide oxidation products **2** and **4**, as well the products of each Cys converted to the cysteic acid and starting material **3**, are observed after two hours (Figure S1).

Having successfully protected the thiol moieties of the EpI sequence, peptide **2** then underwent ion exchange with HBF_4_ resin for the protection of the *N*-terminus and arginine residue as quaternary ammonium salts prior to ruthenium(II)-alkylidene catalysed cyclisation. The metathesis reaction was trialled in DMF with 30 mol% of GII (Figure S2) and the presence of chaotropic salt, LiCl. Gratifyingly, the Ru-catalysed alkylidene metathesis successfully proceeds with 82% conversion to the target product **5** as a mixture of *E*- and *Z*-isomers (Scheme 1). This conversion is consistent with yields observed in the solution phase RCM of another protecting group-free conotoxin sequence [31] and therefore demonstrates the effectiveness of the methods developed for shielding deleterious functional groups within a single substrate.

Desiring an even greener synthesis of dicarba peptides, we investigated thering-closing metathesis of **2**.HBF_4_ in Cyrene™. This dipolar aprotic solvent derived from cellulose is gaining popularity as a DMF replacement and possesses a considerably lower hazard rating [44,45]. RCM of **2**.HBF_4_ with 30 mol% GII catalyst and LiCl in this replacement solvent provides no conversion to product **5**, and only starting material **2** is recovered. Whilst metathesis reactions have been previously achieved in Cyrene™, these were thermodynamically favoured ring-opening metathesis polymerisations using simple monomers [46,47,48]. The cyclisation of the linear peptide may have been inhibited by the higher viscosity of Cyrene™ and/or a deleterious hydrogen bonding network because Cyrene™ is reported to have a lower capacity for accepting hydrogen bonds [49] and hence a lower potential to disrupt aggregation.

Metathesis in water was also attempted, despite known difficulties associated with the need for aqueous-soluble and -active metathesis catalysts. The water-soluble metathesis catalyst developed by Robinson et al. [50] has proved successful on varied amine-bearing substrates protected as their HCl ammonium salts. Peptide **2** was converted to the corresponding HCl ammonium salt using ion exchange resin, maintaining consistency with previously reported metathesis reactions. The RCM was performed on **2**.HCl in water using the water-soluble catalyst (Figure S3) with the addition of LiCl. The reaction was monitored by HPLC over four hours while heating at 80 °C, after which time only starting material **2** was present. The reaction was repeated with the same water-soluble catalyst using metathesis conditions developed by Masuda et al. [51]: 30 mol% catalyst loading, 400 equivalents of MgCl_2_ in aqueous HCl at 60 °C for four hours. Unfortunately, only starting material was recovered again in this case (Catalyst was deemed active by testing the ring-closing metathesis of 2-allyl-pent-4-enylamine hydrochloride, which was achieved in quantitative conversion). The difficulty in achieving metathesis in water is not surprising, as even trace amounts are known to significantly inhibit metathesis yields in much simpler substrates [52]. However, with successful metathesis already achieved in DMF, this result is not detrimental to the overall aim.

With the bicyclic peptide **5** in hand, the reduction of the Met(O) residue to the native Met affords the final target peptide **6**. The reduction was performed by exposing peptide **5** to bromotrimethylsilane, with 2-methyl-2-butene employed as a bromine scavenger (adapted from Beck et al. [53]). The reaction proceeded rapidly with quantitative conversion to the desired peptide **6** within 15 min. Previously reported reduction of sequence-installed Met(O) to Met utilised expensive methionine sulfoxide reductaseenzymes [33,54]. The conditions used herein provide a more facile and cost-effective reduction which can be scaled up and aligned with recombinant production.

## 3. Materials and Methods

### 3.1. General Experimental

Details of instrumentation, solvents and reagents, SPPS procedures and ion exchange resin preparation can be found in the Supplementary Materials linked below.

### 3.2. Compound Synthesis

#### 3.2.1. Synthesis of (S,*Z*)-2-((((9H-Fluoren-9-yl)methoxy)carbonyl)amino)hex-4-enoic Acid

The amino acid was prepared according to Robinson et al. [55] and incorporated into peptide sequences using automated SPPS.

#### 3.2.2. Synthesis of Linear 2,8-*Z*-Crt-10-Met(O)-15-Tyr EpI, **1**

Synthesis of the linear sequence **1** was performed according to the microwave accelerated SPPS procedure described in the Supplementary Materials, using Fmoc-Rink amide resin (0.1 mmol) and Fmoc-L-methionine-D,L-sulfoxide. Following synthesis, the peptide underwent acid-mediated cleavage to provide an off-white solid. A small aliquot of crude peptide **1** was analysed by LC and MS. RP-HPLC (Grace, Columbia, MD, USA, Vydac C18 analytical column, 5–35% buffer B over 30 min): t_R_ = 14.5 min. Mass spectrum (ESI^+^, MeCN, H_2_O, TFA): *m/z* 912.0 [M + 2H]^2+^, ½(C_73_H_113_N_23_O_26_S_3_) theoretical 911.9.

#### 3.2.3. Synthesis of 2,8-*Z*-Crt-10-Met(O)-15-Tyr-*c*[3-16]-cystino EpI, **2**

Buffered aerial oxidation of peptide **1** was carried out according to a procedure described by Clark and colleagues [55]. Crude peptide **1** (103.5 mg, 56.8 μmol) in H_2_O:MeCN (9:1, 40 mL) was added to a stirred solution of 0.1 M NH_4_HCO_3_ (pH 8.5, 350 mL) at room temperature under a constant stream of air. Reaction progress was monitored by RP-HPLC (Grace, Columbia, MD, USA, Vydac C18 analytical column, 5–35% Buffer B over 30 min) and mass spectral analysis. After 21 h, the LC and MS analysis confirmed the formation of the desired peptide **2**. RP-HPLC (Grace, Columbia, MD, USA, Vydac C18 analytical column, 5–35% buffer B over 30 min): t_R_ = 14.0 min. Mass spectrum (ESI^+^, MeCN, H_2_O, TFA): *m/z* 911.0 [M + 2H]^2+^, ½(C_73_H_111_N_23_O_26_S_3_) theoretical 910.9; *m/z* 921.8 [M + H + Na]^2+^, ½(C_73_H_110_NaN_23_O_26_S_3_) theoretical 921.9. The reaction mixture was then acidified to pH 3 with glacial AcOH and purified by preparative RP-HPLC (Grace, Columbia, MD, USA, Vydac C18 preparative column, 5–35% buffer B over 30 min, t_R_ = 17.0 min). Selected fractions were combined and lyophilised to give the target peptide **2** as a colourless solid (44.4 mg, 88% purity). RP-HPLC (Grace, Columbia, MD, USA, Vydac C18 analytical column, 5–35% buffer B over 30 min): t_R_ = 13.4 min. Mass spectrum (ESI^+^, MeCN, H_2_O, TFA): *m/z* 911.1 [M + 2H]^2+^, ½(C_73_H_111_N_23_O_26_S_3_) theoretical 910.9; *m/z* 607.8 [M + 3H]^3+^, ⅓(C_73_H_112_N_23_O_26_S_3_) theoretical 607.6.

#### 3.2.4. Synthesis of Linear 2,8-*Z*-Crt-15-Tyr EpI, **3**

Synthesis of the linear sequence **3** was performed according to the microwave accelerated SPPS procedure described in the Supplementary Materials, using Fmoc-Rink amide resin (0.1 mmol). Following synthesis, the peptide underwent acid-mediated cleavage to provide an off-white solid. A portion of crude peptide **3** (37.0 mg) was purified by RP-HPLC (Grace, Columbia, MD, USA, Vydac C18 preparative column, 5–35% buffer B over 30 min, t_R_ = 20.6 min). Selected fractions were combined and lyophilised to give the target peptide **3** a colourless solid (9.8 mg, 85% purity). RP-HPLC (Grace, Columbia, MD, USA, Vydac C18 analytical column, 5–35% buffer B over 30 min): t_R_ = 17.0 min. Mass spectrum (ESI^+^, MeCN, H_2_O, TFA): *m/z* 1806.7 [M + H]^+^, (C_73_H_112_N_23_O_25_S_3_) theoretical 1806.7; *m/z* 904.0 [M + 2H]^2+^, ½(C_73_H_113_N_23_O_25_S_3_) theoretical 903.9.

#### 3.2.5. Synthesis of 2,8-*Z*-Crt-15-Tyr-*c*[3-16]-cystino EpI, **4**

Buffered aerial oxidation of peptide **3** was carried out according to a procedure described by Clark and colleagues [56]. Crude peptide **3** (84.5 mg, 46.8 μmol) in H_2_O:MeCN 9:1 (30 mL) was added to a stirred solution of 0.1 M NH_4_HCO_3_ pH 8.5 (300 mL) at room temperature under a constant stream of air. Reaction progress was monitored by RP-HPLC (Grace, Columbia, MD, USA, Vydac C18 analytical column, 5–35% buffer B over 30 min) and mass spectral analysis. After 22 h, LC and MS analysis confirmed the formation of the desired peptide **4**. RP-HPLC (Grace, Columbia, MD, USA, Vydac C18 analytical column, 5–35% buffer B over 30 min): t_R_ = 16.1 min. Mass spectrum (ESI^+^, MeCN, H_2_O, TFA): *m/z* 903.4 [M + 2H]^2+^, ½(C_73_H_111_N_23_O_25_S_3_) theoretical 902.9; *m/z* 914.4 [M + H + Na]^2+^, ½(C_73_H_110_NaN_23_O_25_S_3_) theoretical 913.9. The reaction mixture was then acidified to pH 3 with glacial AcOH and purified by preparative RP-HPLC (Grace, Columbia, MD, USA, Vydac C18 preparative column, 5–35% buffer B over 45 min, t_R_ = 25.8 min). Selected fractions were combined and lyophilised to give the target peptide **4** a colourless solid (19.4 mg, >99% purity). RP-HPLC (Grace, Columbia, MD, USA, Vydac C18 analytical column, 5–35% buffer B over 30 min): t_R_ = 16.1 min.

#### 3.2.6. Methionine Oxidation of 2,8-*Z*-Crt-15-Tyr-*c*[3-16]-cystino EpI, **4**, to 2,8-*Z*-Crt-10-Met(O)-15-Tyr-*c*[3-16]-cystino EpI, **2**

The oxidation of M to M(O) was attempted following a procedure adapted from Petitdemange, Lecommandoux et al. [43]. The monocyclic peptide **4**, 2,8-*Z*-Crt-15-Tyr-*c*[3-16]-cystino EpI (0.3 mg) was dissolved in the mixture: 30% H_2_O_2_ (300 μL) + 0.1% AcOH in H_2_O (7 μL) + MQ H_2_O (300 μL) and kept at 0 °C. Reaction progress was monitored by RP-HPLC at t 15 min and 1 h. No significant difference was observed between the two time points. The reaction was quenched by the addition of a few drops of 1 M sodium thiosulfate solution. RP-HPLC (Grace, Columbia, MD, USA, Vydac C18 analytical column, 5–35% buffer B over 30 min): t_R_ = 13.8 min. Mass spectrum (ESI^+^, MeCN, H_2_O, TFA): *m/z* 911.4 [M + 2H]^2+^, ½(C_73_H_111_N_23_O_26_S_3_) theoretical 910.9.

#### 3.2.7. Methionine Oxidation of 2,8-*Z*-Crt-15-Tyr EpI, **3**, to 2,8-*Z*-Crt-10-Met(O)-15-Tyr EpI, **1**

Method A: The oxidation of M to M(O) was attempted following a procedure adapted from Petitdemange, Lecommandoux and colleagues [43]. Purified peptide **3** (0.2 mg) was dissolved in the following mixture: 30% H_2_O_2_ (200 μL) + 0.1% AcOH in H_2_O (5 μL) + MQ H_2_O (200 μL) and kept at 0 °C. Reaction progress was monitored by RP-HPLC at t 15 min, 1 h and 2 h. All time points showed a similar LC profile with the presence of the target peptide **1,** as well as several other oxidation by-products. For the t 2 h time point, RP-HPLC (Grace, Columbia, MD, USA, Vydac C18 analytical column, 5–35% buffer B over 30 min): t_R_ = 13.8 min. Mass spectrum (ESI^+^, MeCN, H_2_O, TFA): *m/z* 911.1 [M + 2H]^2+^, ½(C_73_H_111_N_23_O_26_S_3_) theoretical 910.9. This peak corresponds to peptide **2**, 2,8-*Z*-Crt-10-Met(O)-15-Tyr-*c*[3-16]-cystino EpI. RP-HPLC: t_R_ = 14.5 min. Mass spectrum (ESI^+^, MeCN, H_2_O, TFA): *m/z* 1823.7 [M + H]^+^, (C_73_H_112_N_23_O_26_S_3_) theoretical 1822.7; *m/z* 912.4 [M + 2H]^2+^, ½(C_73_H_113_N_23_O_26_S_3_) theoretical 911.9. This peak is the expected product **1**, 2,8-*Z*-Crt-10-Met(O)-15-Tyr EpI. RP-HPLC: t_R_ = 16.5 min. This peak corresponds to peptide **4,** 2,8-*Z*-Crt-15-Tyr-*c*[3-16]-cystino EpI. RP-HPLC: t_R_ = 17.0 min. This peak is the starting material **3**.

Method B: a modification to Method A was introduced by removing MQ H_2_O from the reaction mixture. Purified peptide **3** (0.2 mg) was dissolved in the following mixture: 30% H_2_O_2_ (300 μL) + 0.1% AcOH in H_2_O (5 μL) and kept at 0 °C. Reaction progress was monitored by RP-HPLC at t 15 min, 1 h and 2 h. Several oxidation by-products were observed (as per Method A), with additional oxidation by-products associated with the oxidation of each cysteine residue to cysteic acid (Figure S1). For the t 1 h time point, RP-HPLC (Grace, Columbia, MD, USA, Vydac C18 analytical column, 5–35% buffer B over 30 min): t_R_ = 12.3 min. Mass spectrum (ESI^+^, MeCN, H_2_O, TFA): *m/z* 936.1 [M + 2H]^2+^, ½(C_73_H_113_N_23_O_29_S_3_) theoretical 935.9. This peak corresponds to 2,8-*Z*-Crt-10-Met(O)-15-Tyr-cysteic acid 3 or 16 EpI. RP-HPLC: t_R_ = 13.0 min. Mass spectrum (ESI^+^, MeCN, H_2_O, TFA): *m/z* 936.1 [M + 2H]^2+^, ½(C_73_H_113_N_23_O_29_S_3_) theoretical 935.9. This peak corresponds to 2,8-*Z*-Crt-10-Met(O)-15-Tyr-cysteic acid 3 or 16 EpI. RP-HPLC: t_R_ = 13.8 min. This peak corresponds to peptide **2**, 2,8-*Z*-Crt-10-Met(O)-15-Tyr-*c*[3-16]-cystino EpI. RP-HPLC: t_R_ = 14.5 min. This peak corresponds to peptide **1**, 2,8-*Z*-Crt-10-Met(O)-15-Tyr EpI. RP-HPLC: t_R_ = 16.5 min. This peak corresponds to peptide **4**, 2,8-*Z*-Crt-15-Tyr-*c*[3-16]-cystino EpI. RP-HPLC: t_R_ = 17.0 min. This peak is the starting material **3**.

#### 3.2.8. Preparation of 2,8-*Z*-Crt-10-Met(O)-15-Tyr-*c*[3-16]-cystino EpI HBF_4_ Salt, **2**.HBF_4_

A sintered column was packed with IRA-400(HBF_4_) resin prepared as described in the Supplementary Materials and swelled in 1:1 H_2_O:MeOH. A solution of monocyclic peptide **2** (9.1 mg, 5.05 µmol) in H_2_O (5 mL) was passed through the resin and collected in a falcon tube. The resin was washed with H_2_O (4 × 5 mL), and the elutes were collected in the falcon tube containing the previously eluted peptide solution. The solution was lyophilised to obtain a colourless solid and quantitative conversion to **2**.HBF_4_ was confirmed with ^19^F-NMR spectroscopy in D_2_O.

#### 3.2.9. Preparation of 2,8-*Z*-Crt-10-Met(O)-15-Tyr-*c*[3-16]-cystino EpI HCl Salt, **2**.HCl

Ion exchange resin IRA-400(Cl) was transferred to a sintered column and swelled in 1:1 H_2_O:MeOH. A solution of peptide **2** (3.5 mg, 1.92 µmol) in H_2_O (3 mL) was passed through the resin and collected in a falcon tube. The resin was then washed with H_2_O (4 × 5 mL), and the elutions were combined with the previously eluted peptide solution. The solution was lyophilised to give the desired ammonium salt **2**.HCl a colourless solid.

#### 3.2.10. Synthesis of *c*[2,8]-Dicarba-10-Met(O)-15-Tyr-*c*[3-16]-cystino EpI, **5**

Method A—in DMF: A Schlenk vessel was charged with monocyclic peptide **2**.HBF_4_ (1.0 mg, 524 nmol), 0.47 mM GII in DMF (350 µL, 30 mol%) (Figure S2) and 0.4 M LiCl in DMF (50 µL) in an inert (N_2_) environment. Solutions had been made using dry, degassed DMF and were freshly sparged with N_2_ prior to use. The vessel was then heated to 100 °C for 4 h under a positive flow of N_2_. After cooling to room temperature, the reaction mixture was concentrated under reduced pressure, taken up in MeOH (~100 µL) and precipitated with Et_2_O (2 mL). Peptide material was collected by centrifugation (1 × 1 min). RP-HPLC and mass spectral analysis of the peptide supported the formation of the required unsaturated carbocycle **5** in 82% conversion as a mixture of *E-* and *Z-*isomers. RP-HPLC (Grace, Columbia, MD, USA, Vydac C18 analytical column, 5–35% buffer B over 30 min): t_R_ = 9.8 and 10.2 min. Mass spectrum (ESI^+^, MeCN:H_2_O:TFA): *m/z* 883.1 [M + 2H]^2+^, ½(C_69_H_103_N_23_O_26_S_3_) theoretical 882.8. The reaction mixture was then purified by analytical RP-HPLC (Grace, Columbia, MD, USA, Vydac C18 column, 5–35% buffer B over 30 min, t_R_ = 9.8 and 10.2 min). Coeluting isomeric fractions were combined and lyophilised to give the target peptide **5** a colourless solid (0.3 mg, >99% purity). High resolution mass spectrum (ESI^+^, MeCN:H_2_O:TFA): *m/z* 882.8298 [M + 2H]^2+^, ½(C_69_H_103_N_23_O_26_S_3_) theoretical 882.8298. ^1^H NMR (600 MHz, D_2_O) δ 7.20–7.11 (m, 2H), 6.89–6.82 (m, 2H), 5.72–5.54 (m, 2H), 5.20–5.14 (m, 1H), 5.11–4.95 (m, 1H), 4.68–4.60 (m, 1H), 4.60–4.49 (m, 1H), 4.49–4.40 (m, 2H), 4.39–4.23 (m, 2H), 4.05–3.69 (m, 7H), 3.66–3.43 (m, 1H), 3.33–3.16 (m, 4H), 3.16–2.64 (m, 13H), 2.64–2.52 (m, 1H), 2.46–2.37 (m, 1H), 2.33–2.21 (m, 2H), 2.20–2.11 (m, 1H), 2.08 (t, *J* = 6.8 Hz, 1H), 2.05–1.95 (m, 9H), 1.95–1.83 (m, 1H), 1.83–1.72 (m, 1H), and 1.71–1.55 (m, 3H). A total of 42 protons were not observed.

Method B—in Cyrene™: A Schlenk vessel was charged with linear peptide **2**.HBF_4_ (2.5 mg, 1.31 µmol), 0.4M LiCl in DMF (50µL) and 0.47 mM GII in Cyrene™ (880 µL, 30 mol%) (Figure S2) in an inert (N_2_) environment. The solution had been made using dry, degassed Cyrene™ and was freshly sparged with N_2_ prior to use. The vessel was then heated to 100 °C for 4 h under a positive flow of N_2_. After cooling to room temperature, the reaction mixture was cooled on ice and precipitated with ice-cold Et_2_O (7 mL). Peptide material was collected by centrifugation (6000 rpm × 6 min). RP-HPLC and mass spectral analysis of the peptide showed no conversion to desired carbocycle **5**.

Method C—in water: Water soluble catalyst was prepared according to the preparation by Robinson and colleagues [50]. Linear peptide **2**.HCl (2.5 mg, 1.31 µmol) and LiCl (10 mg) were added to a solution of catalyst **7** (270 µg, 30 mol%) (Figure S3) in water (2 mL). The vessel was then heated to 80 °C for 4 h. An aliquot of the reaction mixture was injected for RP-HPLC. RP-HPLC and mass spectral analysis of the peptide showed no conversion to desired carbocycle **5**.

Method D—in water: Metathesis in water was attempted using conditions from Yoshiya and colleagues [51]. Water soluble catalyst was prepared according to the preparation by Robinson and colleagues [50]. The linear peptide **2**.HCl (2.5 mg, 1.31 µmol) and MgCl_2_·6H_2_O (53 mg, 400 equiv.) were added to a solution of catalyst **7** (270 µg, 30 mol%) (Figure S3) in 0.1 M HCl (aq.) (2 mL). The vessel was then heated to 60 °C for 4 h. An aliquot of the reaction mixture was injected for RP-HPLC. RP-HPLC and mass spectral analysis of the peptide showed no conversion to desired carbocycle **5**.

#### 3.2.11. Synthesis of *c*[2,8]-Dicarba-15-Tyr-*c*[3-16]-cystino EpI, **6**

Dicarba peptide **5** (0.3 mg, 170 nmol) was dissolved in TFA (0.5 mL). To the peptide solution, 2-methyl-2-butene (11 µL, 103 µmol) and bromotrimethylsilane were added (26 µL, 197 µmol). The reaction was shaken at room temperature for 15 min before removing TFA under flow of N_2_. The remaining residue was taken up in ice-cold Et_2_O (1 mL), and the precipitated peptide was collected by centrifugation (1 × 1 min). RP-HPLC and mass spectral analysis of the peptide supported the formation of the methionine-containing dicarba peptide **6** in quantitative conversion as a mixture of *E-* and *Z-*isomers. RP-HPLC (Grace, Columbia, MD, USA, Vydac C18 analytical column, 5–35% buffer B over 30 min): t_R_ = 11.4 and 11.6 min. Mass spectrum (ESI^+^, MeCN:H_2_O:TFA): *m/z* 875.3 [M + 2H]^2+^, (C_69_H_103_N_23_O_25_S_3_) theoretical 874.8. The reaction mixture was purified by analytical RP-HPLC (Grace, Columbia, MD, USA, Vydac C18 column, 5–35% buffer B over 30 min, t_R_ = 11.4 and 11.6 min). Coeluting isomer fractions were combined and lyophilised to give the target peptide **6** a colourless solid (0.1 mg, 95% purity).

## 4. Conclusions

In the current work, the α-conotoxin EpI sequence from *Conus episcopatus* was used as an exemplar to showcase reversible protection methodologies for problematic coordinating functionalities, including amines, thiols and thioethers. Ring-closing metathesis of off-resin and unprotected peptides was successful and generated the two target dicarba peptide analogues, *cis-* and *trans*-[2,8]-dicarba EpI **6**. The scalable methodology described herein aligns with growing interest in the recombinant production of peptides of industrial relevance which importantly interfaces with the genetic incorporation of metathesis-active residues, such as allylglycine and crotyl glycine, into linear peptide precursors [57,58]. Future work will assess the in vitro activity of the novel *c*[2,8]-dicarba EpI isomers on both GABA_B_ and nACh receptors. With the loop I sequence of EpI being identical to other analgesic α-conotoxins, it is anticipated that the dicarba EpI analogues will show a similar activity trend [22,23]. In conclusion, the target dicarba EpI α-conotoxin derivatives were generated via a generic SPPS and protecting group-free metathesis approach, which focused on reducing the use of unsafe organic solvents, elimination of sidechain protecting groups, telescoped reactions and exploitation of mild and chemoselective oxidation conditions.

## Data Availability

Not applicable.

## Supplementary Materials

The following supporting information can be downloaded at: https://www.mdpi.com/article/10.3390/md21070390/s1, Figure S1: LC traces of the Met to Met(O) oxidation of peptide **3** using Method B, and (B) LC traces of the Met to Met(O) oxidation of peptide **4** to peptide **2**. The * in (A) denotes peptides with the expected Met(O) as well as the oxidation of either Cys 2 or Cys 8 to cysteic acid.; Figure S2: Structure of Grubbs 2nd Generation **GII** catalyst (1,3-bis(2,4,6-trimethylphenyl)-2-imidazolidinylidene) dichloro (phenylmethylene) (tricyclohexylphosphine) ruthenium(II); Figure S3: Structure of water soluble catalyst 7 from Robinson et al.; General Experimental Information and Supporting Spectra. References [50,59] are cited in the supplementary materials.
