# Supplementary material for: Synthesis of Cystine-Stabilised Dicarba Conotoxin EpI: Ring-Closing Metathesis of Sidechain Deprotected, Sulfide-Rich Sequences
Source: Mar Drugs. 2023 Jun 29;21(7):390. doi: 10.3390/md21070390 (PMC10381330; doi:10.3390/md21070390)
Supplement: Supplementary file 1 [file marinedrugs-21-00390-s001.zip › marinedrugs-2453008-supplementary.pdf]

Supporting Information

Synthesis of cystine-stabilised dicarba Conotoxin EpI: Ring-closing metathesis of sidechain deprotected, sulfide-rich sequences

Author names

Amy L. Thomson, Andrea J. Robinson, Alessia Belgi *

School of Chemistry, Monash University, Clayton, Victoria 3800, Australia

* Corresponding Author: alessia.belgi@monash.edu

Table of Contents

General Experimental Information 2
Figure S1 5
Figure S2 6
Figure S3 6
Supplementary Spectra 7
References 19

General Experimental Information

General Considerations

Manipulation of organometallic compounds was performed using standard Schlenk techniques under an atmosphere of dry nitrogen.

Instrumentation

Low resolution electrospray ionisation (ESI) mass spectra were recorded a Waters Micromass ZQ Mass Spectrometer as solutions in specified solvents. Spectra were recorded in positive mode (ESI^+).

Fluorine nuclear magnetic resonance (^{19}F-NMR) spectra were recorded on Bruker BACS400 or EXPRESS400 operating at 400 MHz as solutions of deuterated solvents as specified.

Reverse-phase high performance liquid chromatography (RP-HPLC) was performed on Agilent 1200 series instruments. For analytical experiments, the instrument was equipped with photodiode array (PDA) detection (controlled by ChemStation software) and a manual injector (100 µL loop volume). Experiments were carried out on a Vydac C18 analytical column (4.6 mm x 250 mm, 5 µm) at a flow rate of 1.5 mL min^{-1}. For preparative runs, the instrument used multivariable wavelength (MVW) detection (controlled by ChemStation software) and an Agilent unit injector (2 mL loop volume). Experiments were carried out on a Vydac C18 preparative column (22 mm x 250 mm, 10 µm) at a flow rate of 10 mL min^{-1}. The solvent systems were buffer A, 0.1% aqueous TFA; buffer B, 0.1% TFA in MeCN.

Proton (^1H) NMR spectra spectra were recorded on a Bruker AV600 operating at 600 MHz.

Chemical shifts (δ) are expressed in parts per million (ppm), and proton spectra were referenced to the residual deuterium oxide solvent peak (δ 4.79 ppm). Each resonance was assigned according to the following convention: chemical shift; multiplicity; observed coupling constants (*J* Hz); number of protons. Splitting patterns are expressed as singlet (s), doublet (d), triplet (t), quartet (q), multiplet (m).

Solvents and Reagents

Dimethylformamide (DMF), dichloromethane (CH_2Cl_2), diethyl ether (Et_2O), acetonitrile (MeCN), methanol (MeOH), trifluoroacetic acid (TFA), tetrafluoroboric acid, sodium bicarbonate, hydrogen peroxide, deuterium oxide, lithium chloride, magnesium chloride, Cyrene™, 2-methyl-2-butene, bromotrimethylsilane (TMSBr), Amberlite IRA400(Cl), Amberlite IRA400(OH), and (1,3-bis(2,4,6-trimethylphenyl)-2-imidazolidinylidene)dichloro(phenylmethylene)(tricyclohexylphosphine)ruthenium (**GII**) (Supplementary data) were supplied by Sigma-Aldrich (St. Louis, Missouri, USA). Triisopropylsilane

(TIPS), *N,N*-diisopropylethylamine (DIPEA) and *N*-methyl-2-pyrrolidone (NMP) were supplied by Oakwood Chemical (South Carolina, USA). Acetic acid (AcOH) and hydrochloric acid were used as supplied by Ajax Finechem (New South Wales, Australia). Fmoc protected amino acids, Rink amide resin and 1-[bis(dimethylamino)methylene]-1*H*-1,2,3-triazolo[4,5-*b*]pyridinium 3-oxide hexafluorophosphate (HATU) were supplied by Mimotopes (Melbourne, Australia).

General Procedure for Solid Phase Peptide Synthesis (SPPS)

Resin wash and filtering steps were aided by the use of a Visiprep SPE DL 24-port model vacuum manifold as supplied by Supelco. Cleavage mixtures were shaken on a KS125 basic KA elliptical shaker supplied by Labortechnik at 400 motions per minute.

Automated microwave-accelerated SPPS was carried out using a CEM Liberty-Discover synthesizer, involving the flow of dissolved reagents from external nitrogen pressurized bottles to a resin-containing microwave reactor vessel fitted with a porous filter. Coupling and deprotection reactions were carried out within this vessel and were aided by microwave energy. Each reagent delivery, wash, and evacuation step was carried out according to the automated protocols of the instrument (controlled by PepDriver software). In a 50 mL centrifuge tube, resin was swollen with DMF:CH_2Cl_2 (1:1; 10 mL, 1 x 60 min) and connected to the Liberty resin manifold. The Fmoc-amino acids (0.2 M in DMF), activators (0.5 M HATU in DMF), activator base (2 M DIPEA in NMP), and deprotection agent (20% v/v piperidine in DMF) were solubilized in an appropriate volume of specified solvent as calculated by the PepDriver software program. The default microwave conditions used in the synthesis of each linear peptide included: initial deprotection (36 W, 37 °C, 2 min), deprotection (45 W, 75 °C, 10 min), pre-activation (0 W, 25 °C, 2 min), and coupling (25 W, 75 °C, 10 min), or initial deprotection (40 W, 75 °C, 0.5 min), deprotection (40 W, 75 °C, 3 min), and coupling (20 W, 75 °C, 5 min). After sequence completion, the resin-bound peptides were automatically returned to the Liberty resin manifold as a suspension in DMF: CH_2Cl_2 (1:1) and filtered through fritted plastic syringes (5 mL) for acid-mediated cleavage.

TFA Cleavage Procedure

A small aliquot of resin-bound peptide (approximately 3 mg) was suspended in cleavage solution (1 mL; 95:2.5:2.5; TFA:TIPS:H_2O) and shaken gently for 2 h. The mixture was filtered through a fritted syringe and the beads rinsed with TFA (1 × 0.2 mL). The filtrate was concentrated under a constant stream of air, and the resultant oil was induced to precipitate in ice-cold Et_2O (1 mL). Cleaved peptides

were collected by centrifugation (3 × 5 min) and dried for analysis by analytical RP-HPLC and mass spectrometry. For full-scale resin cleavages, 10 mL of cleavage solution was used, and after 4 h, the resin was rinsed with TFA (3 × 1 mL). The filtrate was concentrated under a constant stream of air, and the resultant oil was induced to precipitate in ice-cold Et_2O (30−35 mL). Collection by centrifugation was carried out over 5 × 6 min spin times. Cleaved peptides were collected by centrifugation at a speed of 6000 rpm on a Hermle Z200A centrifuge supplied by Medos, or at a speed of 6000 rpm on a TMC-1 mini centrifuge supplied by Thermoline.

General Procedure for preparing HBF_4 counter ion exchange resin

Counter ion exchange resin was prepared according to a modified procedure outlined by Alcalde and Dinarès.[1] In a sintered funnel, wet strongly basic ion exchange Amberlite IRA-400(OH) (5 g) was washed with H_2O (50 mL) and H_2O:MeOH (1:1) (50 mL). A solution of 1% HBF_4 in 1:1 H_2O:MeOH was slowly passed through the resin until the eluent had the same pH value as the original acid solution. The resin was then transferred to a round-bottom flask and stirred in the 1% HBF_4 solution (50 mL) for a further 4 h at room temperature. The resin was filtered and washed generously with both H_2O:MeOH (1:1) (200 mL) and H_2O (500 mL) and allowed to air dry.

Figure S1

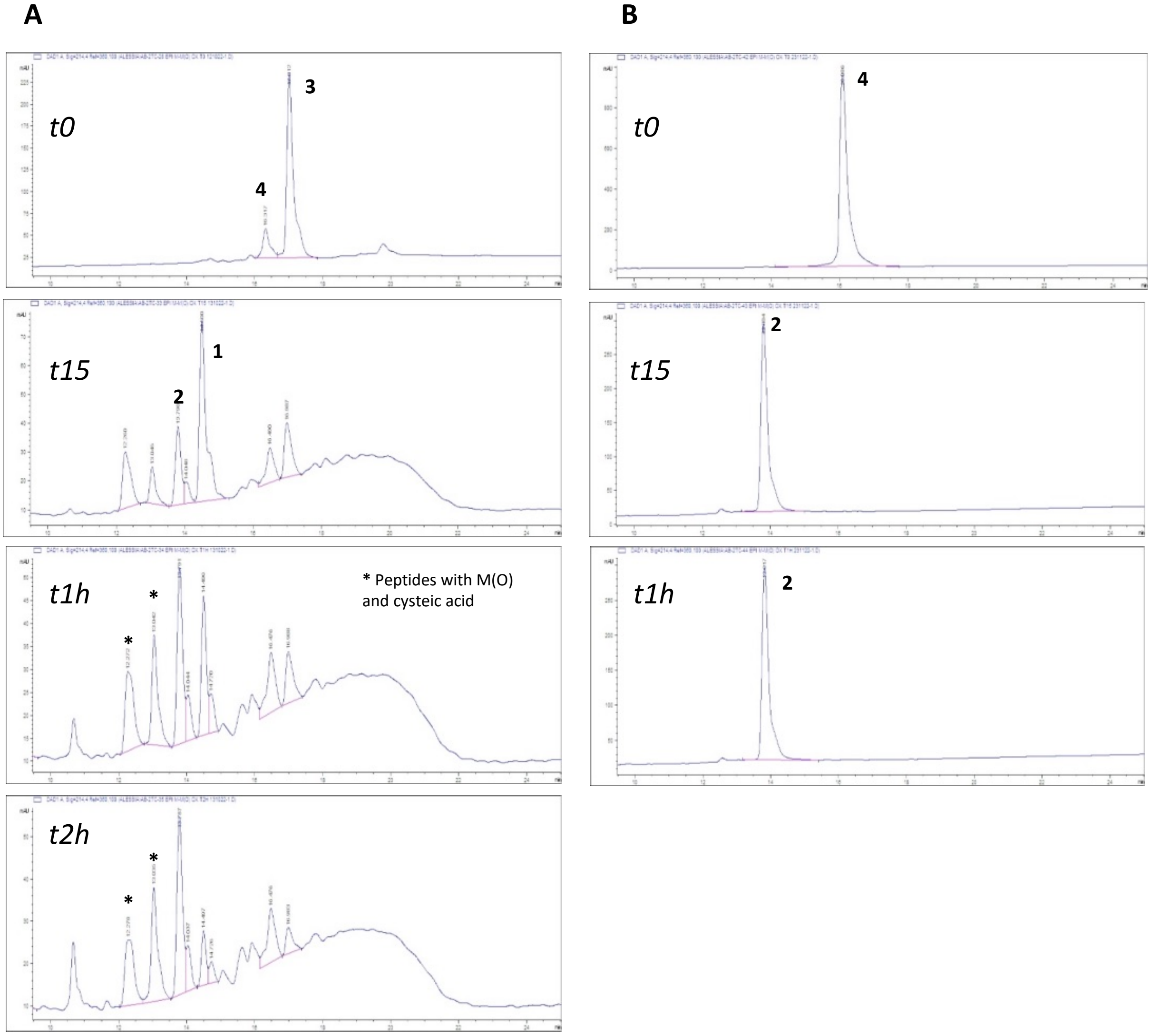

***Figure S1.** LC traces of the Met to Met(O) oxidation of peptide **3** using Method B, and (**B**) LC traces of the Met to Met(O) oxidation of peptide **4** to peptide **2**. The * in (**A**) denotes peptides with the expected Met(O) as well as the oxidation of either Cys 2 or Cys 8 to cysteic acid.*

Figure S2

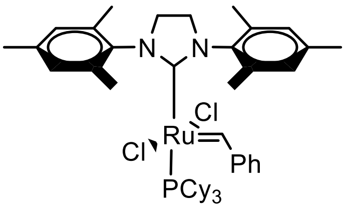

*Figure S2. Structure of Grubbs 2nd Generation **GII** catalyst (1,3-bis(2,4,6-trimethylphenyl)-2-imidazolidinylidene)dichloro(phenylmethylene)(tricyclohexylphosphine)ruthenium(II).*

Figure S3

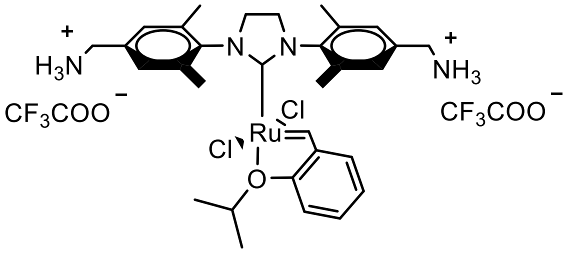

Figure S3. Structure of water soluble catalyst 7 from Robinson et al. *[2].*

Supplementary Spectra

Linear 2,8-*Z*-Crt-10-Met(O)-15-Tyr EpI, 1

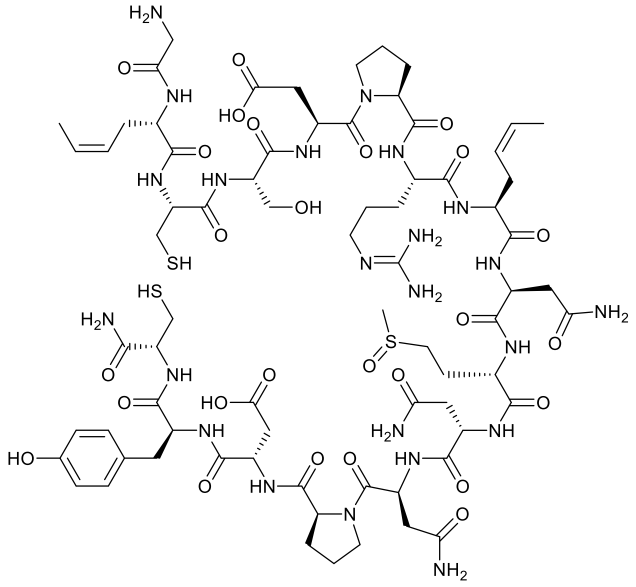

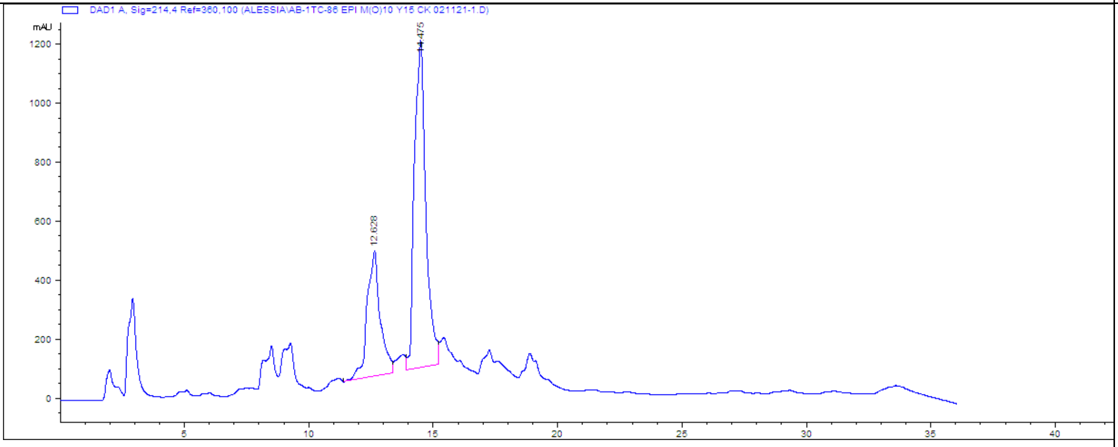

Crude peptide (45% purity)

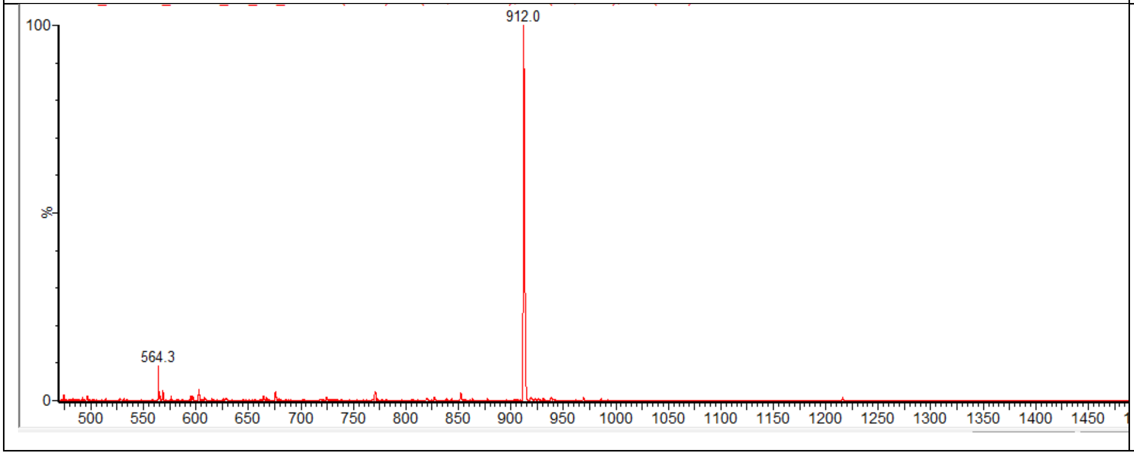

2,8-*Z*-Crt-10-Met(O)-15-Tyr-c[3-16]-cystino EpI, 2

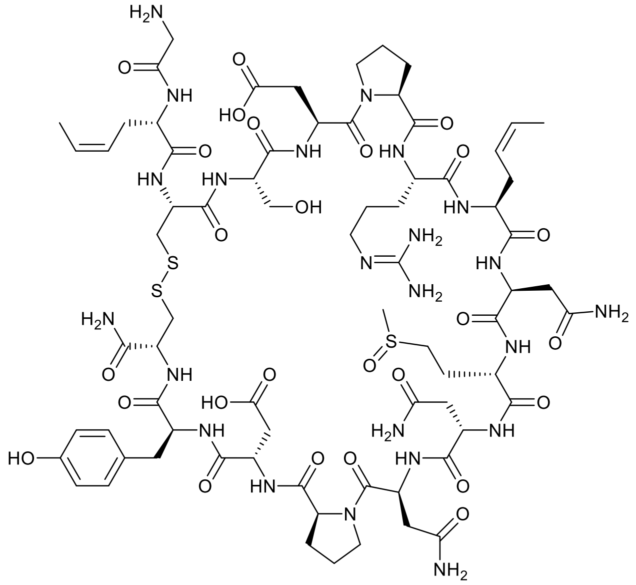

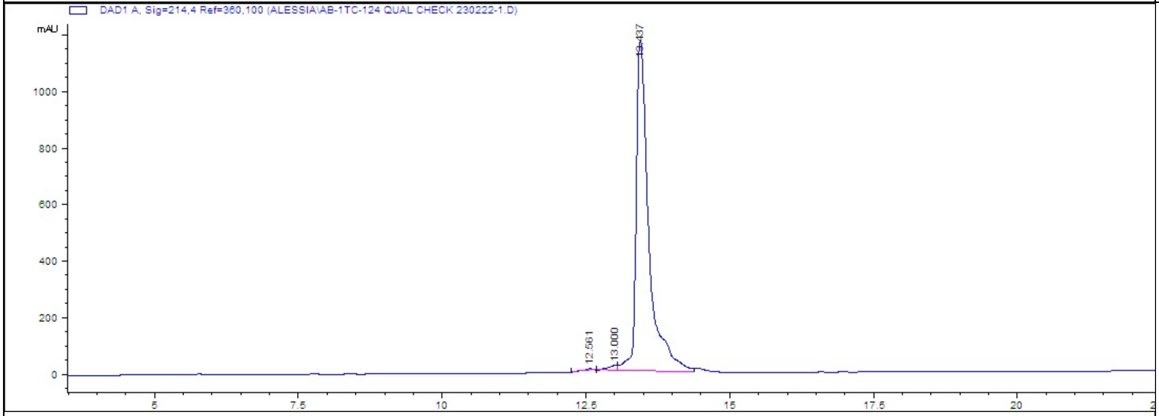

Purified peptide post S-S oxidation (88% purity)

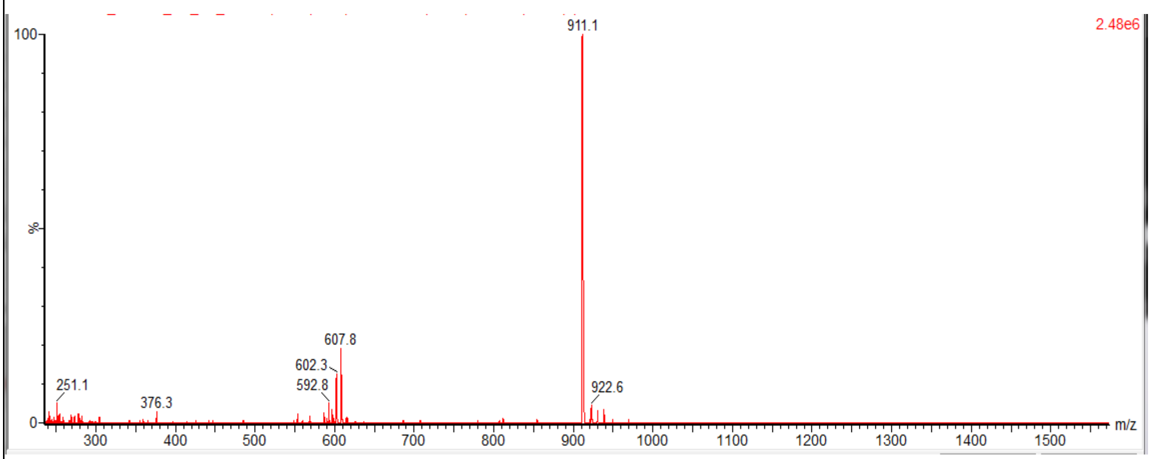

Linear 2,8-*Z*-Crt-15-Tyr EpI, 3

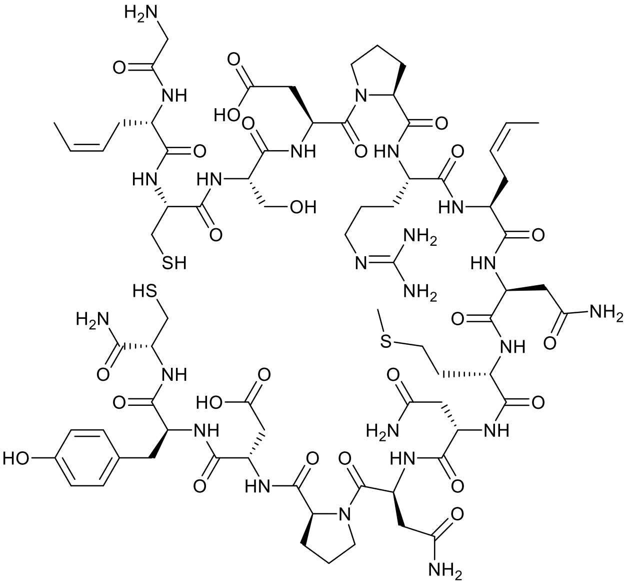

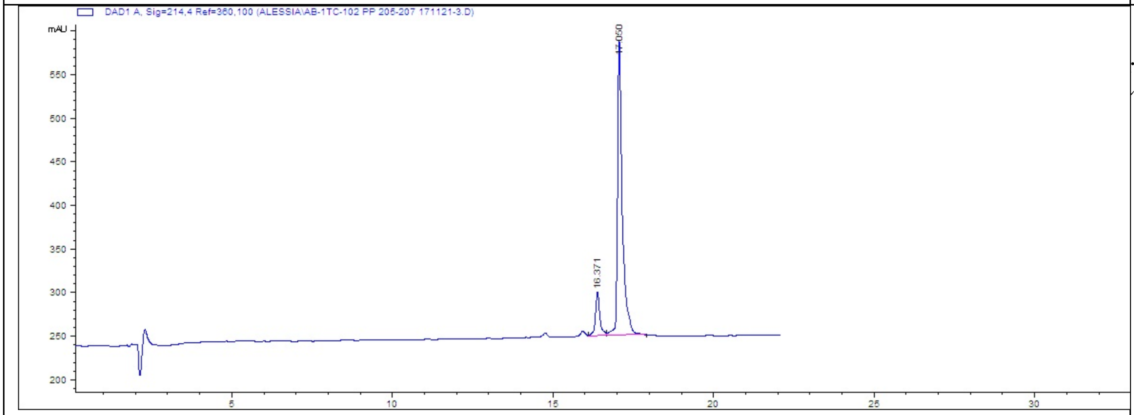

Partially purified peptide (85%

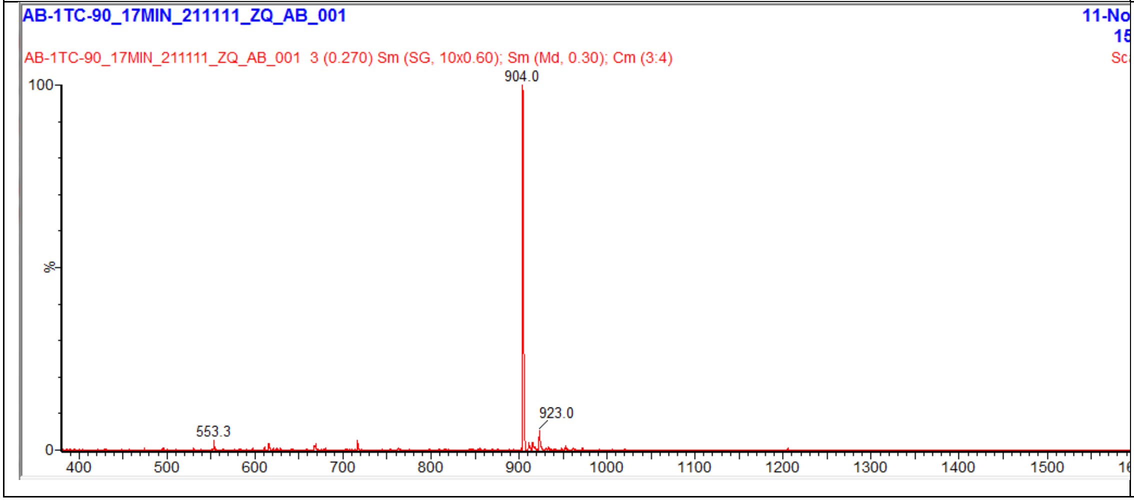

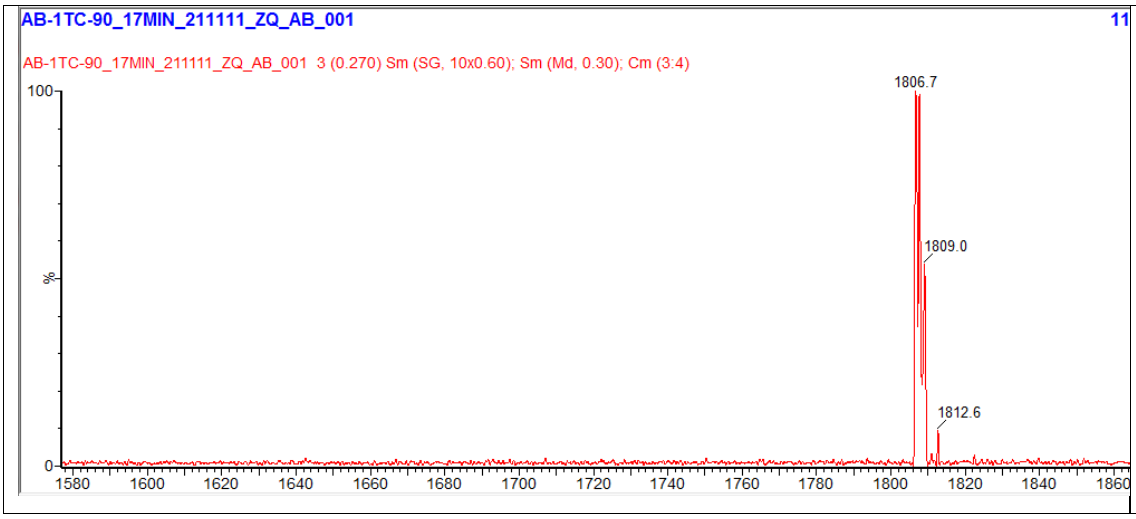

2,8-*Z*-Crt-15-Tyr-c[3-16]-cystino EpI, 4

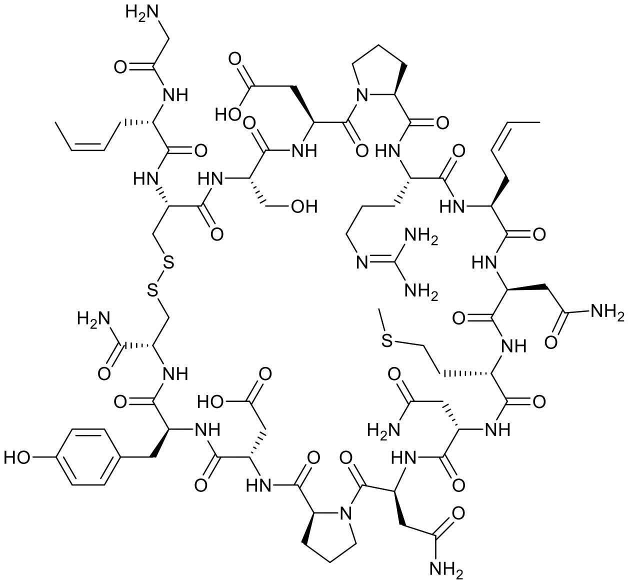

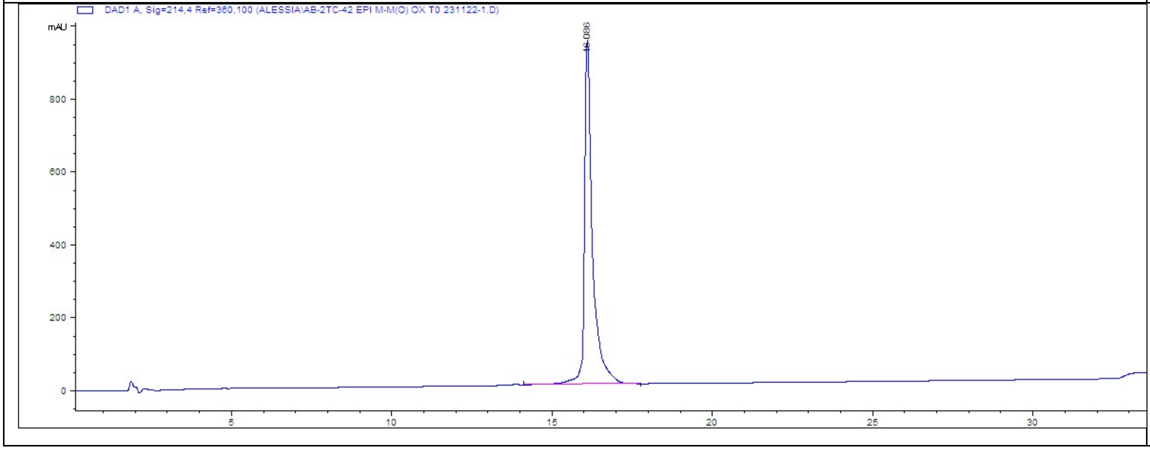

Purified peptide post S-S oxidation (>99% purity)

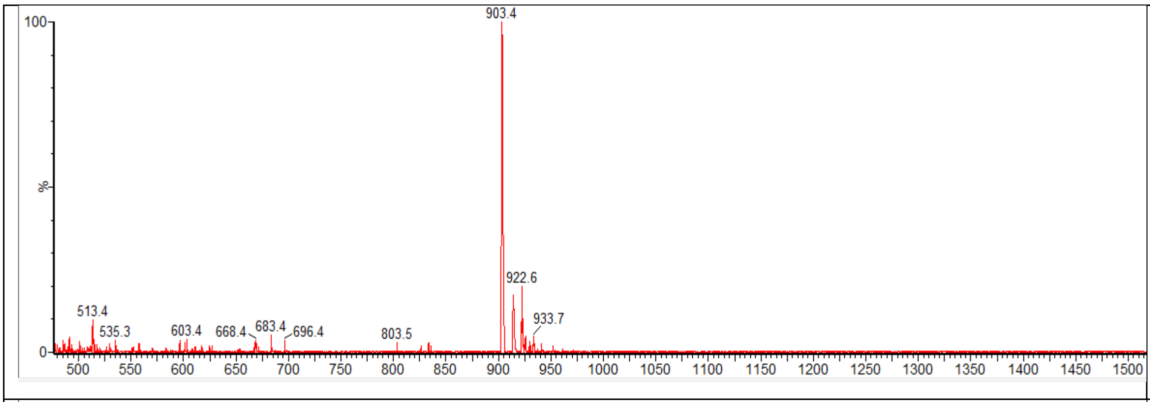

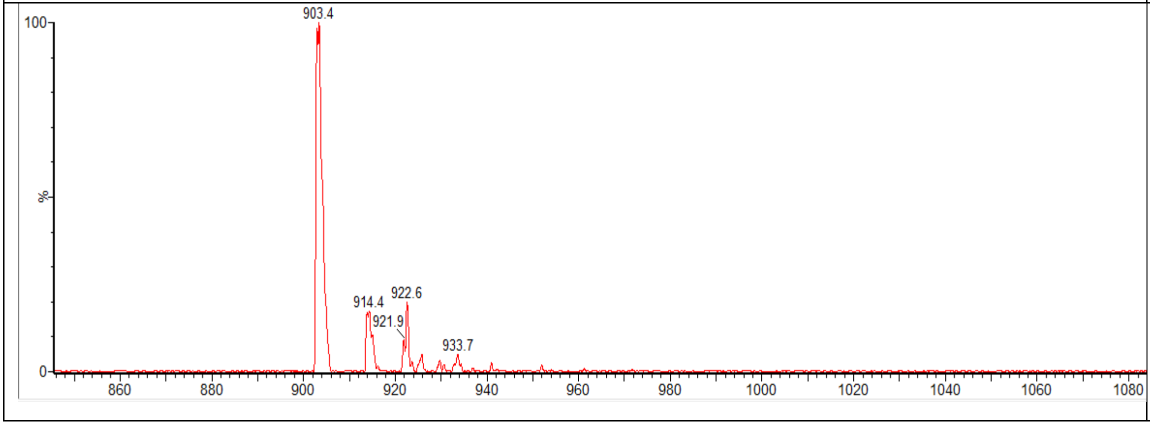

Methionine Oxidation of 2,8-*Z*-Crt-15-Tyr-c[3-16]-cystino EpI, 4, to 2,8-*Z*-Crt-10-Met(O)-15-Tyr-c[3-16]-cystino EpI, 2	
t15 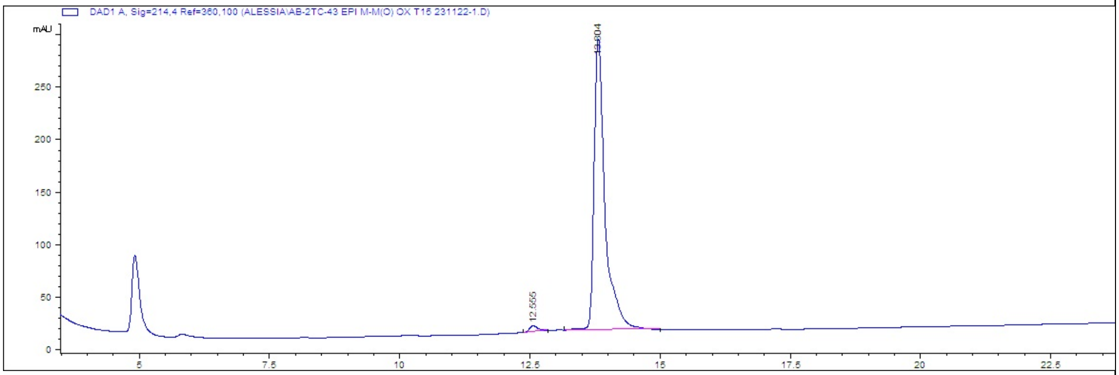	Crude reaction mix trace
t1h 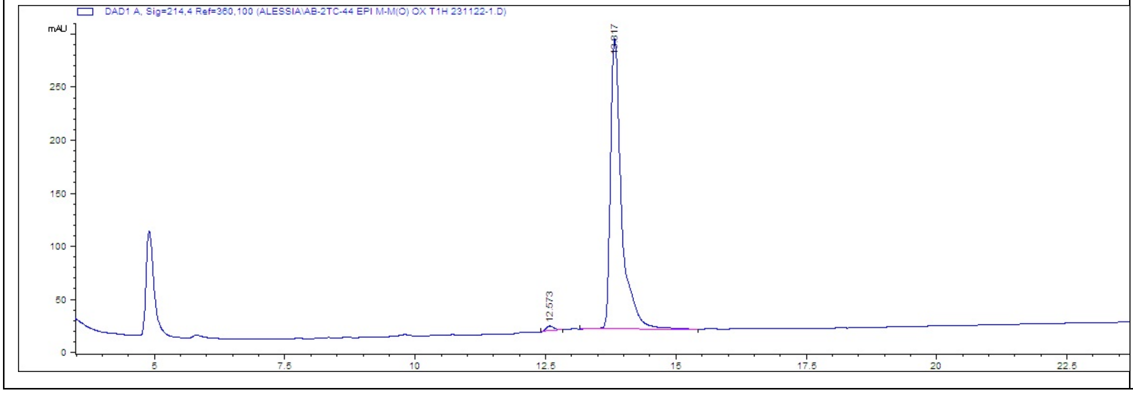	Crude reaction mix trace

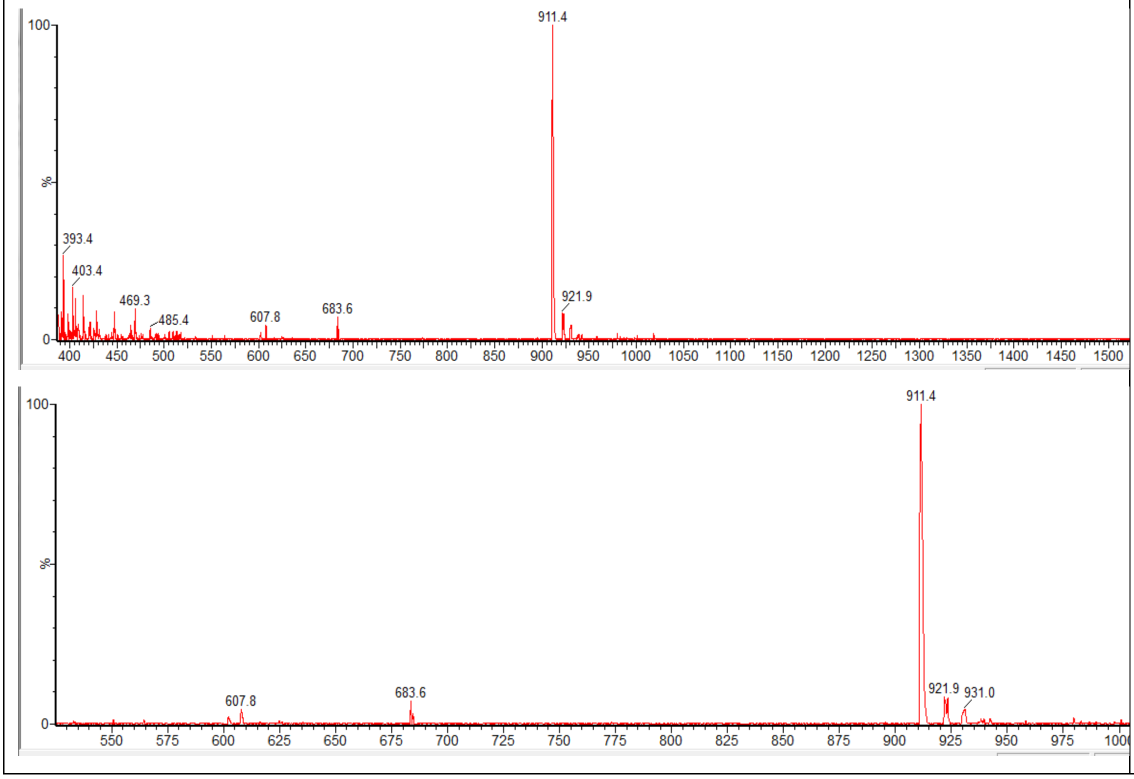	

**Methionine Oxidation of 2,8-*Z*-Crt-15-Tyr EpI, 3, to 2,8-*Z*-Crt-10-Met(O)-15-Tyr EpI, 1**	
**Method A**	
t15 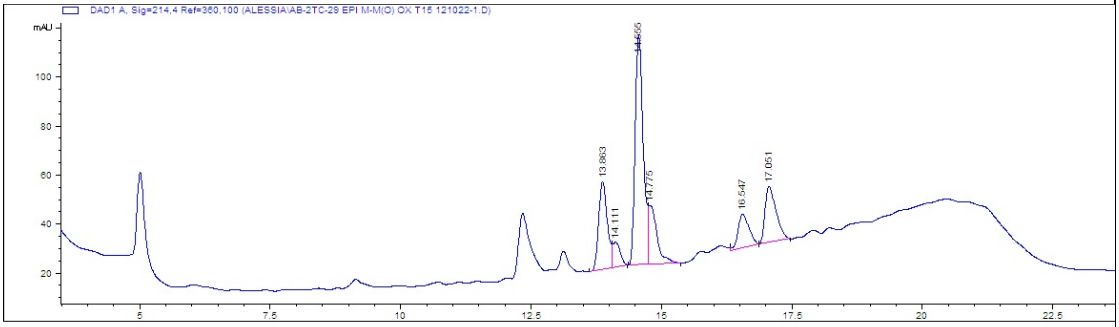	Crude reaction mix trace
t1h 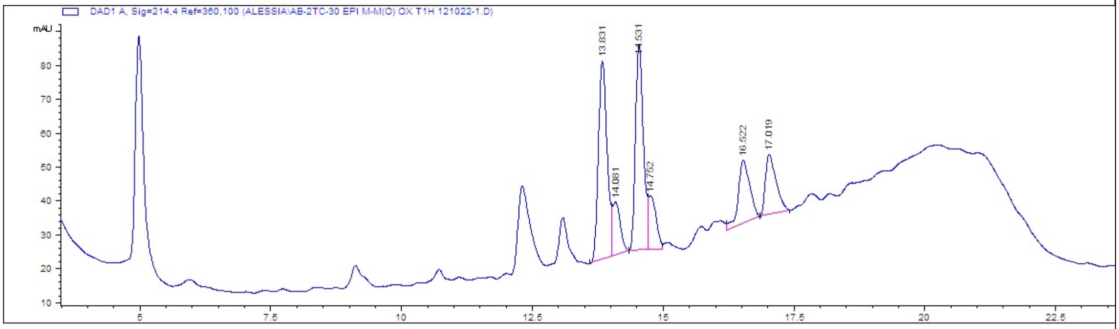	Crude reaction mix trace

t2h

Crude reaction mix trace

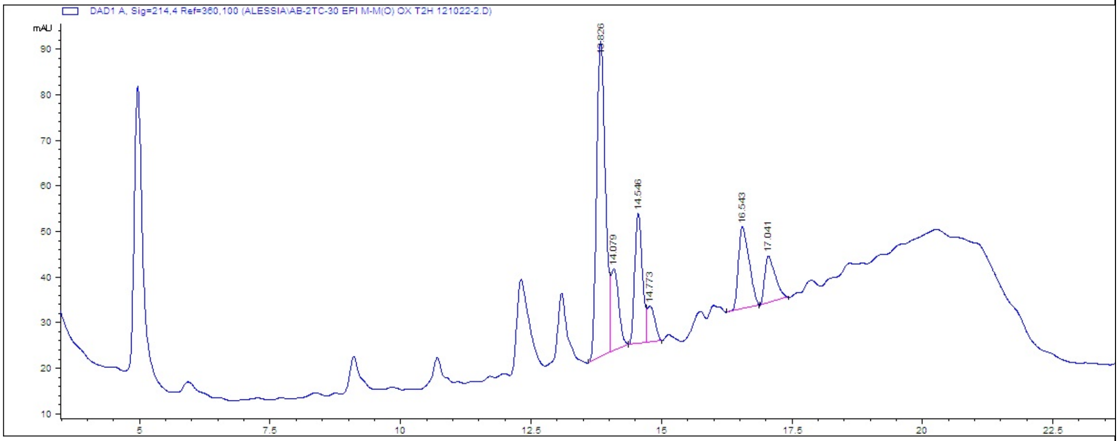

13.8 min

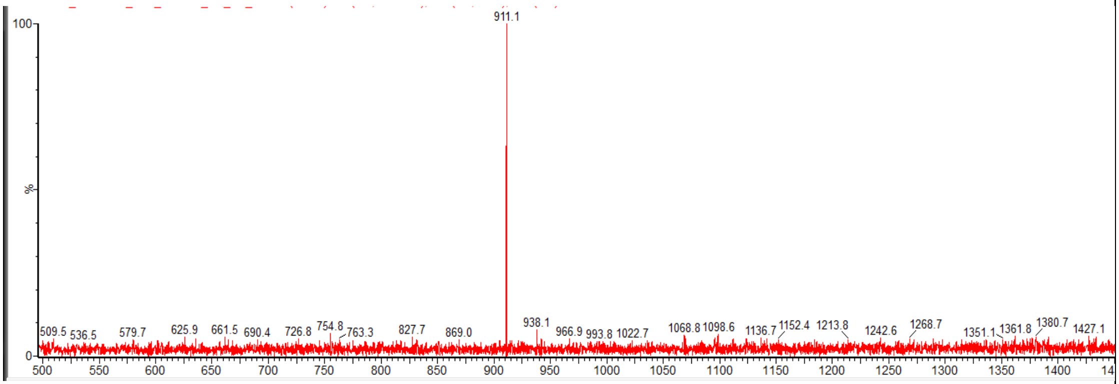

14.5 min

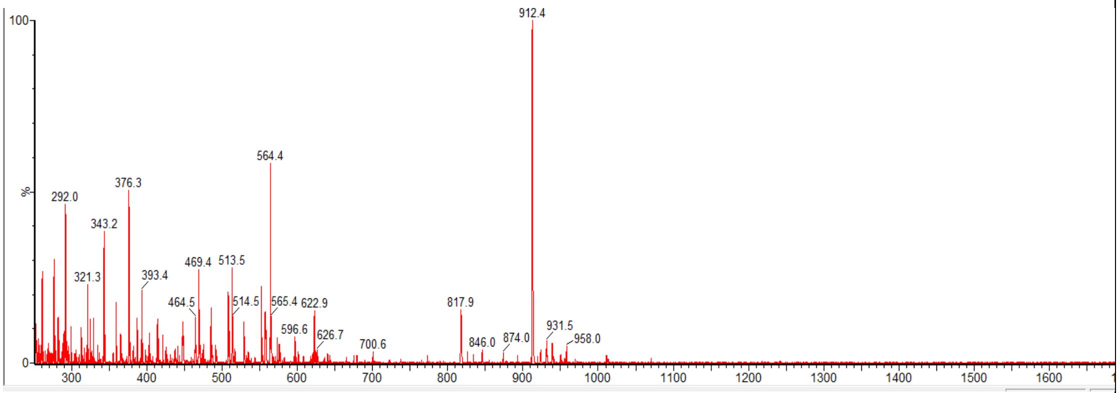

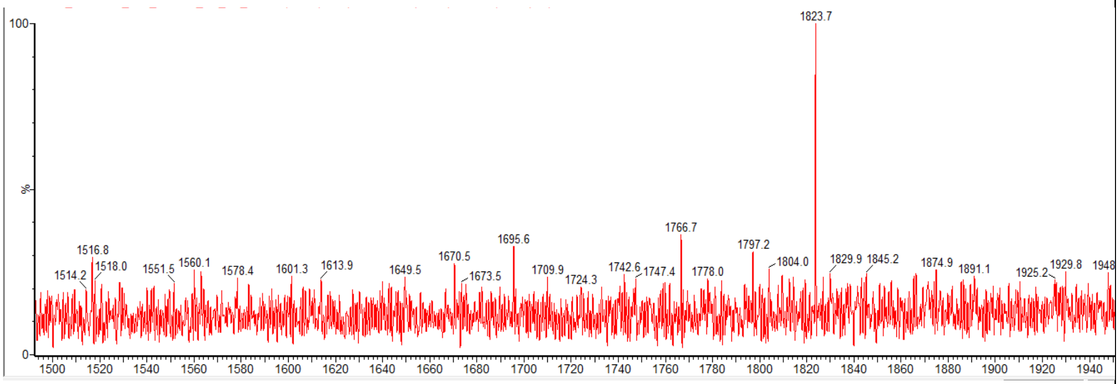

Method B

t15

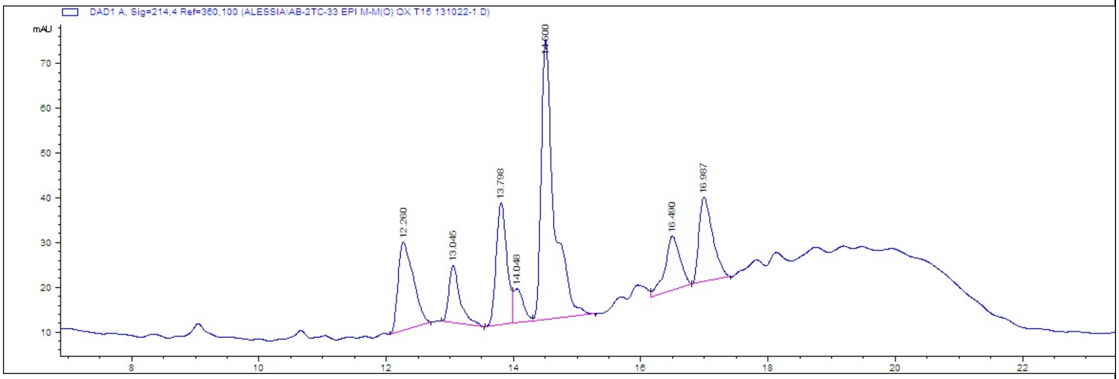

Crude reaction mix trace

t1h

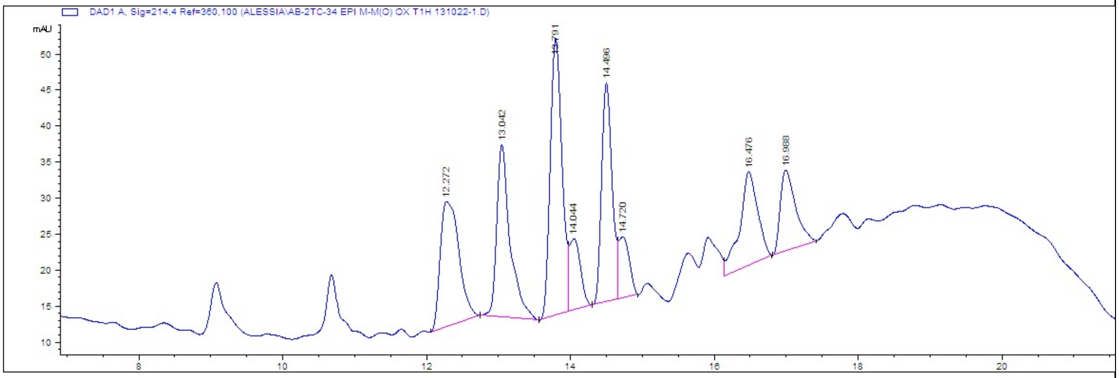

Crude reaction mix trace

t2h

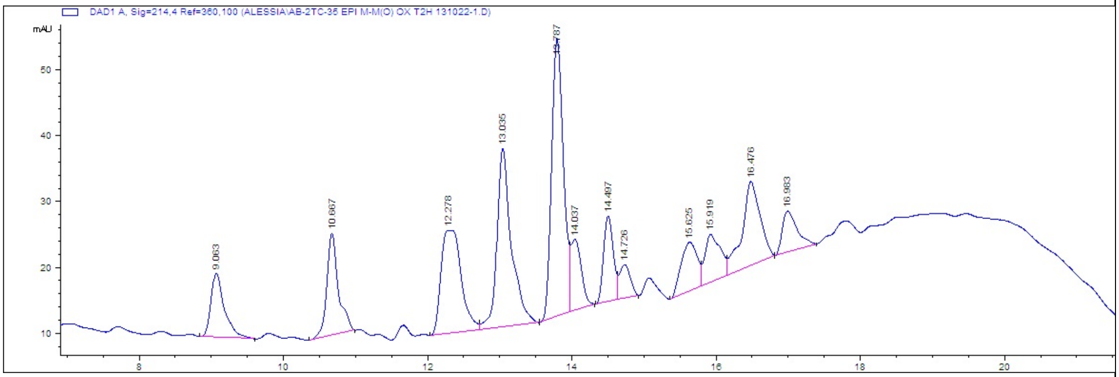

Crude reaction mix trace

12.3 min

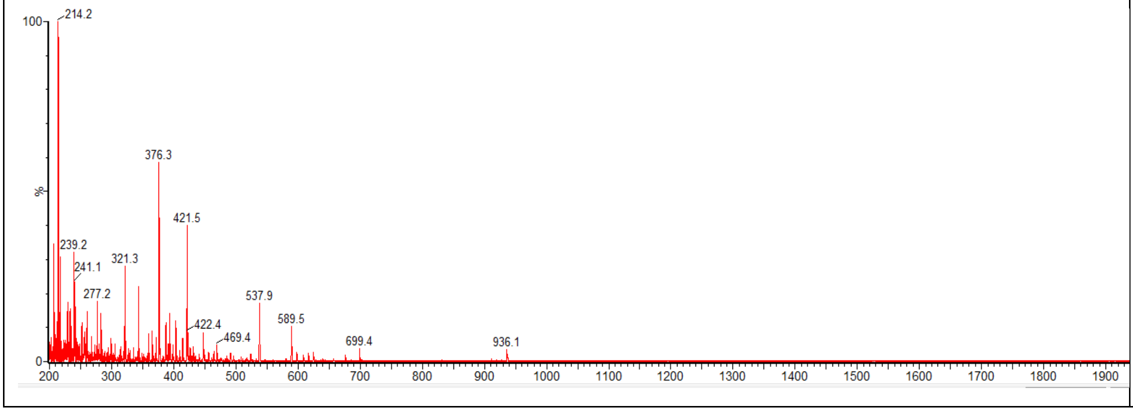

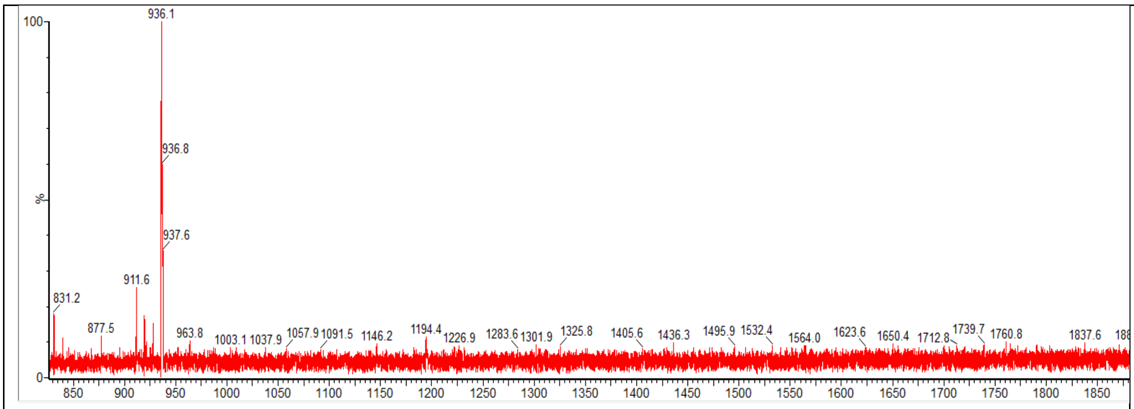
100
%
0
936.1
936.8
937.6
911.6
831.2
877.5
963.8
1003.1
1037.9
1057.9
1091.5
1146.2
1194.4
1226.9
1283.6
1301.9
1325.8
1405.6
1436.3
1495.9
1532.4
1564.0
1623.6
1650.4
1712.8
1739.7
1760.8
1837.6
850
900
950
1000
1050
1100
1150
1200
1250
1300
1350
1400
1450
1500
1550
1600
1650
1700
1750
1800
1850

13.0 min

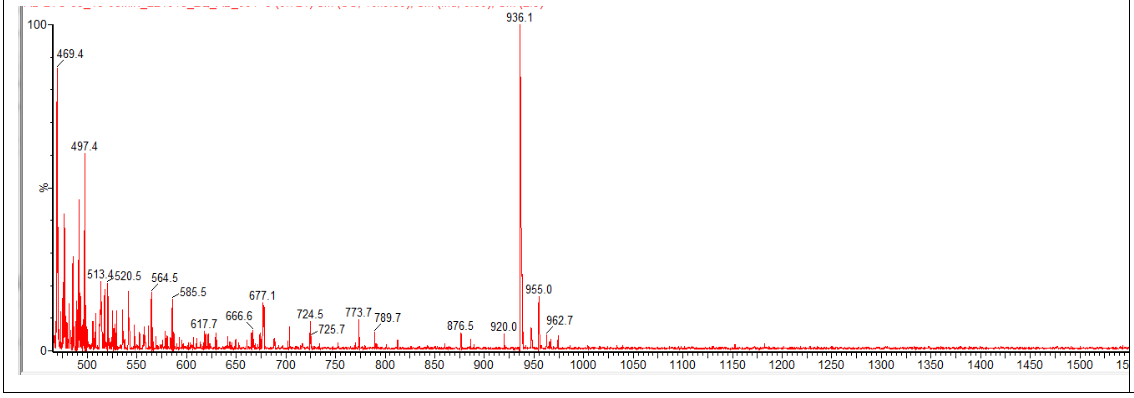
100
%
0
936.1
469.4
497.4
513.4
520.5
564.5
585.5
617.7
666.6
677.1
724.5
725.7
773.7
789.7
876.5
920.0
955.0
962.7
500
550
600
650
700
750
800
850
900
950
1000
1050
1100
1150
1200
1250
1300
1350
1400
1450
1500

2,8-Dicarba-10-Met(O)-15-Tyr EpI, 5

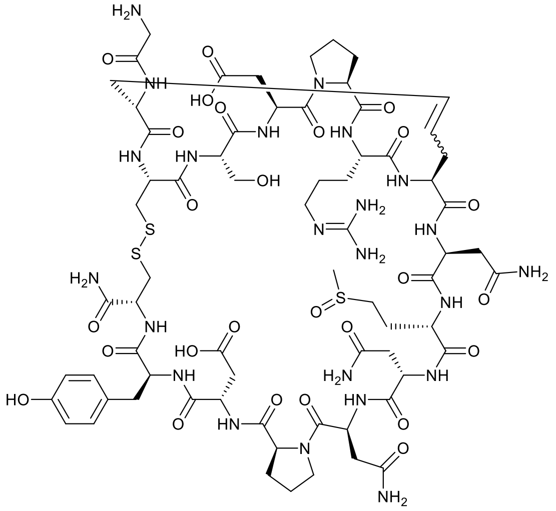

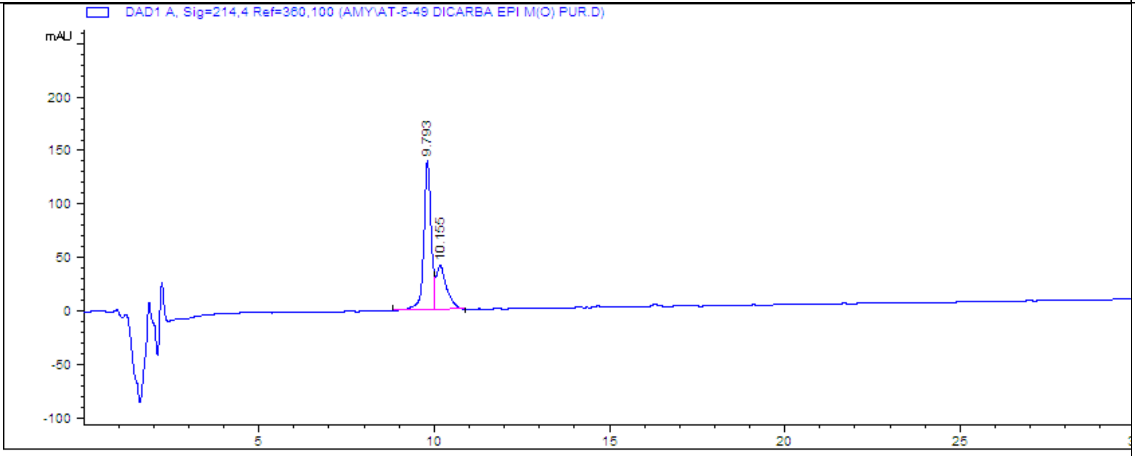

Purified peptide isomers (>99% purity)

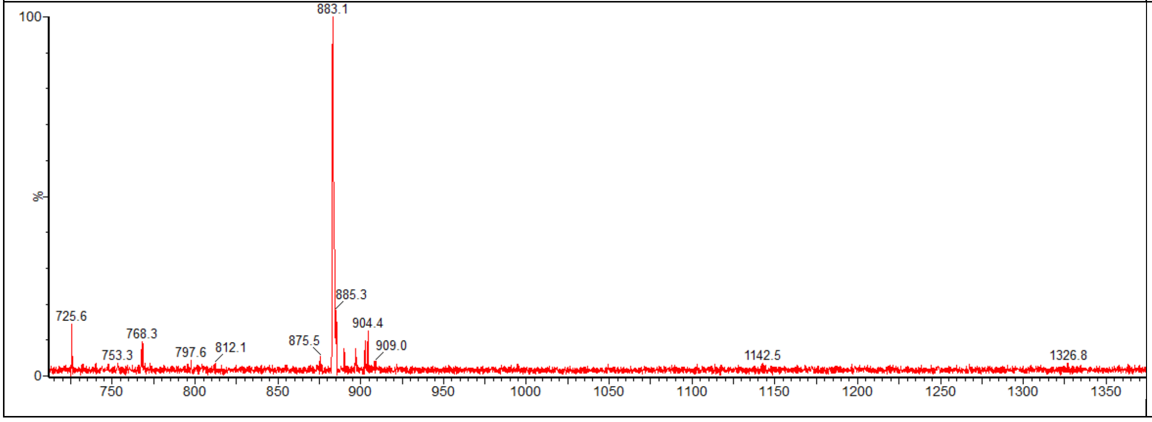

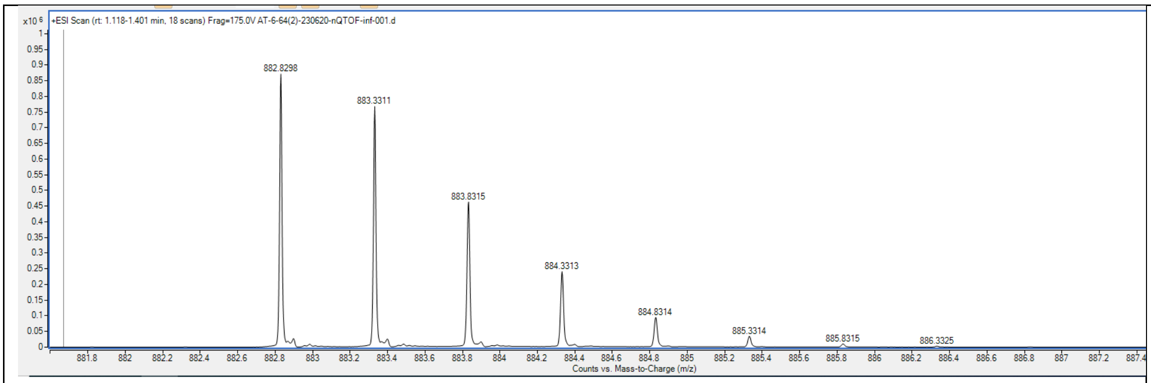
+ESI Scan (rt: 1.118-1.401 min, 18 scans) Frag=175.0V AT-6-64(2)-230620-nQTOF-inf-001.d
882.8298
883.3311
883.8315
884.3313
884.8314
885.3314
885.8315
886.3325
Counts vs. Mass-to-Charge (m/z)

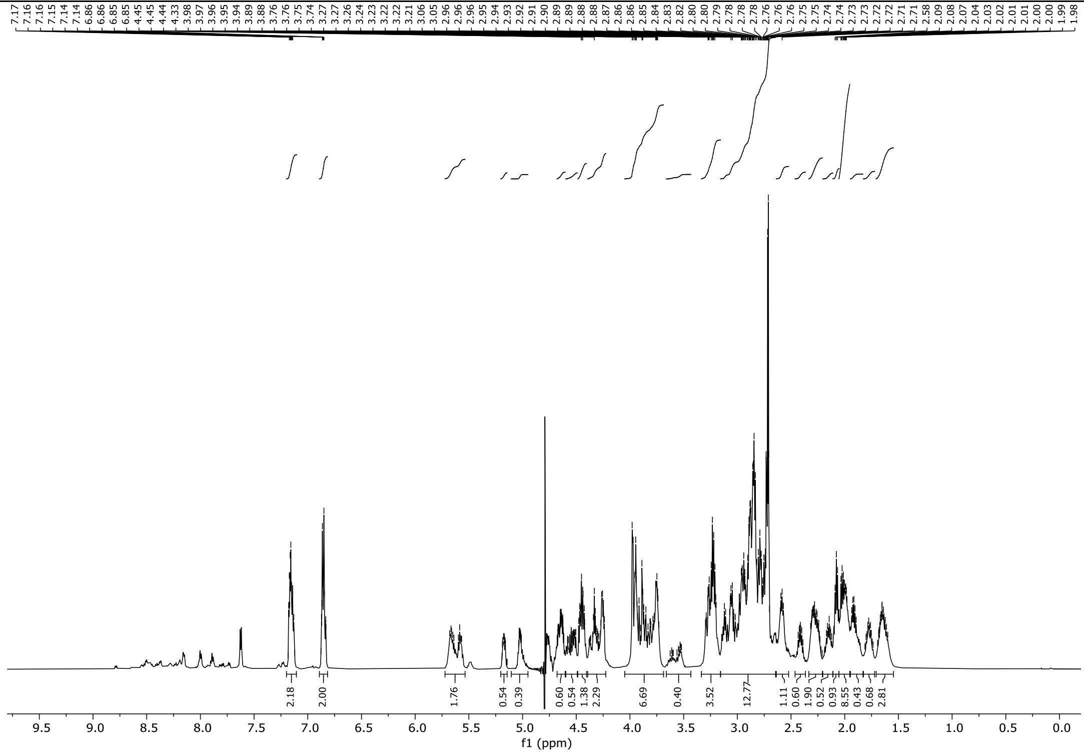
f1 (ppm)

2,8-Dicarba-15-Tyr EpI, 6

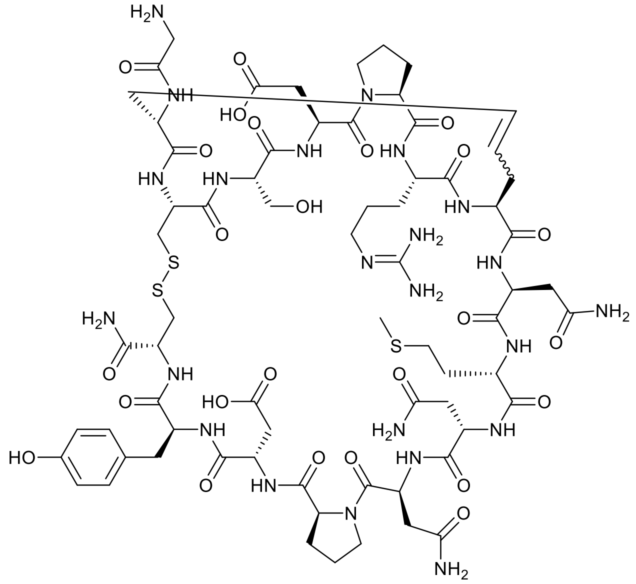

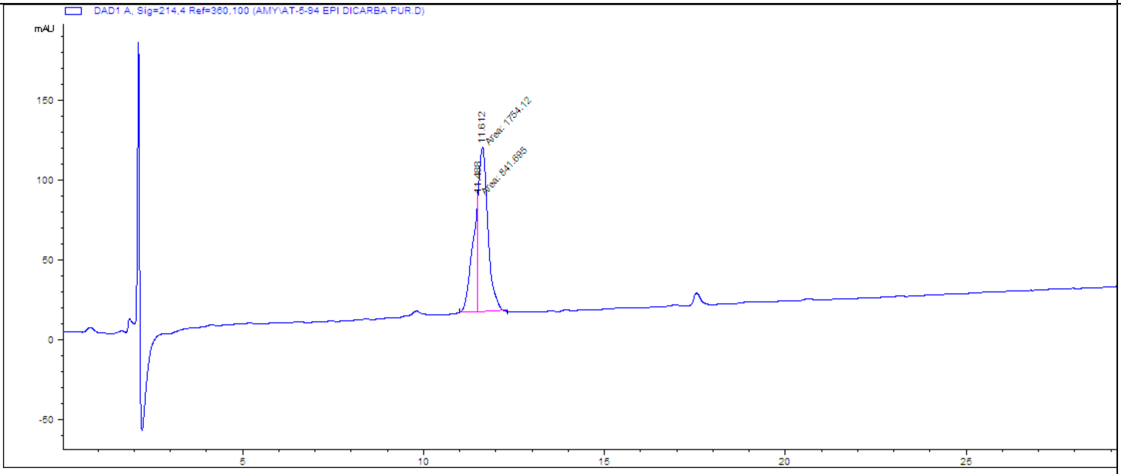

Purified peptide isomers (95% purity)

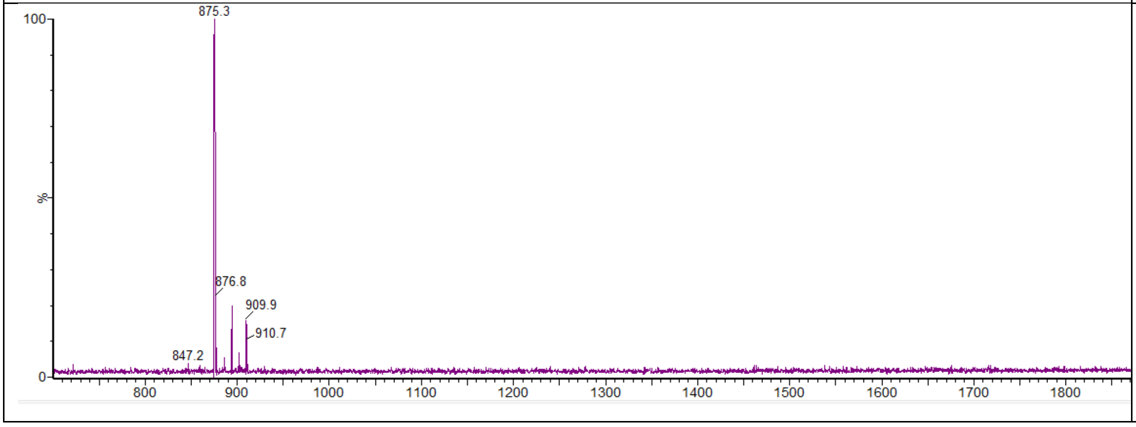

References

1. Alcalde, E.; Dinares, I.; Ibañez, A.; Mesquida, N., A simple halide-to-anion exchange method for heteroaromatic salts and ionic liquids. *Molecules* **2012**, *17*(4), 4007-4027.

2. Wang, Z. J.; Jackson, W. R.; Robinson, A. J., A simple and practical preparation of an efficient water soluble olefin metathesis catalyst. *Green Chemistry* **2015**, *17*(6), 3407-3414.
